# Physical activity and fertility

**DOI:** 10.1123/jpah.2022-0487

**Published:** 2023-05-05

**Authors:** Alison K. Brinson, Shana G. da Silva, Kathryn R. Hesketh, Kelly R. Evenson

**Affiliations:** University of North Carolina—Chapel Hill, Chapel Hill, NC, USA; Federal University of Fronteira Sul, Passo Fundo, RS, Brazil University of North Carolina—Chapel Hill, Chapel Hill, NC, USA; University of North Carolina—Chapel Hill, Chapel Hill, NC, USA UCL Great Ormond Street Institute of Child Health, London, UK MRC Epidemiology Unit, University of Cambridge, Cambridge, UK; University of North Carolina—Chapel Hill, Chapel Hill, NC, USA

## Abstract

**Background:**

Before pregnancy is recognized, ovulation, fertilization, and implantation must all occur. Physical activity and sedentary behavior may impact pregnancy success by altering each or all of these processes. The aim of this review was to review the association between physical activity and sedentary behavior with spontaneous female and male fertility.

**Method:**

PubMed/MEDLINE, Web of Science, CINAHL, SPORTDiscus, and Embase were searched from inception to August 9, 2021. Eligible studies included randomized controlled trials or observational studies, published in English, describing an association between physical activity or sedentary behavior (exposures) and spontaneous fertility (outcome) among females or males.

**Results:**

Thirty-four studies from 31 unique populations were included in this review (12 cross-sectional studies, 10 cohort studies, 6 case control studies, 5 randomized controlled trials, and 1 case cohort study). Of the 25 studies among females, the majority identified mixed results (n=11) or no association (n=9) between physical activity and female fertility. Seven studies reported on female fertility and sedentary behavior, 2 found sedentary behavior was associated with decreased female fertility. Of the 11 studies among males, most of the studies (n=6) found physical activity was associated with increased male fertility. Two of the studies reported on male fertility and sedentary behavior, neither identified an association.

**Conclusions:**

The association between spontaneous fertility and physical activity in both males and females remains unclear, while the association with sedentary behavior remains largely unexplored.

## Introduction

Fertility is defined as the capacity to establish a clinical pregnancy.^1,2^ For a pregnancy to be recognized, ovulation, fertilization, and implantation all must occur.^3^ Physical activity and sedentary behavior may impact fertility and subsequent pregnancy success by altering any or all of these processes. Among women, increased physical activity and decreased sedentary behavior may improve fertility through maintenance of body weight and hormone levels necessary for increased likelihood of conception.^4^ In contrast, high levels of physical activity could inhibit ovulation, leading to decreased fertility.^5^ The American College of Obstetrics and Gynecology (ACOG) recommend females planning to initiate pregnancy achieve a minimum of 150 minutes of moderate physical activity per week.^6^ However, these guidelines do not provide further information on how the type, intensity, or duration of physical activity may alter fertility status.

Much less is known about the biologic impact of physical activity on male fertility. Physical activity may influence fertility through the alteration of key semen parameters including concentration, motility, and morphology.^7,8^ Moderate physical activity appears to positively influence male fertility status, while high intensity activity may be detrimental.^8^ Sedentary behavior and fertility have yet to be addressed in any of the clinical or national guidelines. This is necessary given the increased research linking sedentary behavior with adverse health outcomes, independent of physical activity.^9^

Since fertility treatments can be costly, and fertility problems can contribute to negative emotional and psychological outcomes,^10^ it seems prudent to explore the associations between fertility and modifiable behaviors like physical activity and sedentary behavior. We therefore systematically reviewed the literature in order to summarize the association between physical activity and sedentary behavior with spontaneous (i.e., non-assisted) female and male fertility.

## Materials and Methods

This systematic review was conducted according to the Preferred Reporting Items for Systematic Reviews and Meta Analyses (PRISMA) guidelines.^11,12^ This review was registered with International Prospective Register of Systematic Reviews (PROSPERO) and assigned #CRD42016041797.^13,14^

### Search Strategy

We performed electronic literature searches in PubMed/MEDLINE, Web of Science, Embase, CINAHL, and SPORTDiscus from inception through August 9, 2021 (Appendix 1). We also searched the reference lists of each included study. The screening process of titles and abstracts was conducted independently by two researchers (shared between the authors) and disagreements were resolved by consensus. Results of the searches were exported to a citation manager (EndNote version X8.2) for removal of duplicates.

### Study Selection

To be included in this review a study needed to be published in English and report an association between physical activity or sedentary behavior exposure and an outcome of spontaneous fertility. We included all types of physical activities (i.e., leisure, occupational, transportation, household) and sedentary behaviors (i.e., sitting) and included both device-based and self-report measures. Studies needed to focus on the outcome of spontaneous fertility, including fecundity (e.g., capacity to conceive and produce offspring) or fecundability (e.g., probability of achieving pregnancy in a single menstrual cycle).^15^ Studies that reported on male and female fertility were included, regardless of the fertility background of the participants and their partners (e.g., a previous infertility diagnosis), participant age, or participant body mass index (BMI).

We included observational studies (cross-sectional, cohort, case-cohort, and case-control) if physical activity or sedentary behavior was identified as one of the exposures of interest. We also included randomized controlled trials if (i) the intervention focused solely on physical activity or sedentary behavior, (ii) at least one outcome measure included physical activity or sedentary behavior, and (iii) a control group was included. For example, the study by Homan et al.^18^ was not included because the intervention targeted multiple health behaviors and not solely physical activity or sedentary behavior.

We excluded studies exploring behavior change among women preparing for infertility treatment^19^ since we wanted to specifically assess the impact of physical activity or sedentary behavior on spontaneous fertility. For outcomes, we excluded studies with an assisted fertility outcome (e.g., in vitro fertilization), or studies focused on reproductive disorders that may be risk factors for infertility, such as endometriosis, polycystic ovary syndrome, or uterine fibroids. Moreover, studies that focused on component causes of infertility (i.e., structural problems, endocrine disorders, diminished ovarian reserve, or autoimmune disorder), used ovarian markers (i.e., Anti-Mullerian hormone, follicular stimulating hormone, or antral follicle count), or used semen/sperm parameters (i.e., motility, count, volume, or concentration) were not included.

### Data Extraction

Data from selected studies were screened by two reviewers separately. Each study was read in full, and the following information was extracted: study location, years of data collection, study design, sample size for enrollment and analysis, sample inclusion and/or exclusion criteria, recruitment methods, definition and ascertainment methods of exposure and outcome, main results, and confounding variables considered. A second reviewer checked the extraction and disagreements were resolved by consensus. There were several differences in how the exposures were measured and defined. Therefore, we were unable to perform meta-analytic analyses to assess for heterogeneity or publication bias.

### Study Quality Assessment

Quality of cross-sectional, case cohort, case control, and cohort studies was described using the 14-item National Institutes of Health Study Quality Assessment Tool for Observational Cohort and Cross-Sectional Studies.^20^ Quality of randomized controlled trials, was described using the 14-item National Institutes of Health Study Quality Assessment Tool for Controlled Intervention Studies.^21^ Both assessment tools were modified for the purpose of this review (Supplementary Tables 1, 2, & 3). In recognition that objective quality assessment tools treat each threat to validity equally,^22^ we did not provide a total score for each study. Instead, we used the quality assessment results to focus on the specific threats to validity identified across included studies. Two reviewers assessed study quality and all disagreements were resolved by consensus.

## Results

### Search Results

**Figure 1:**
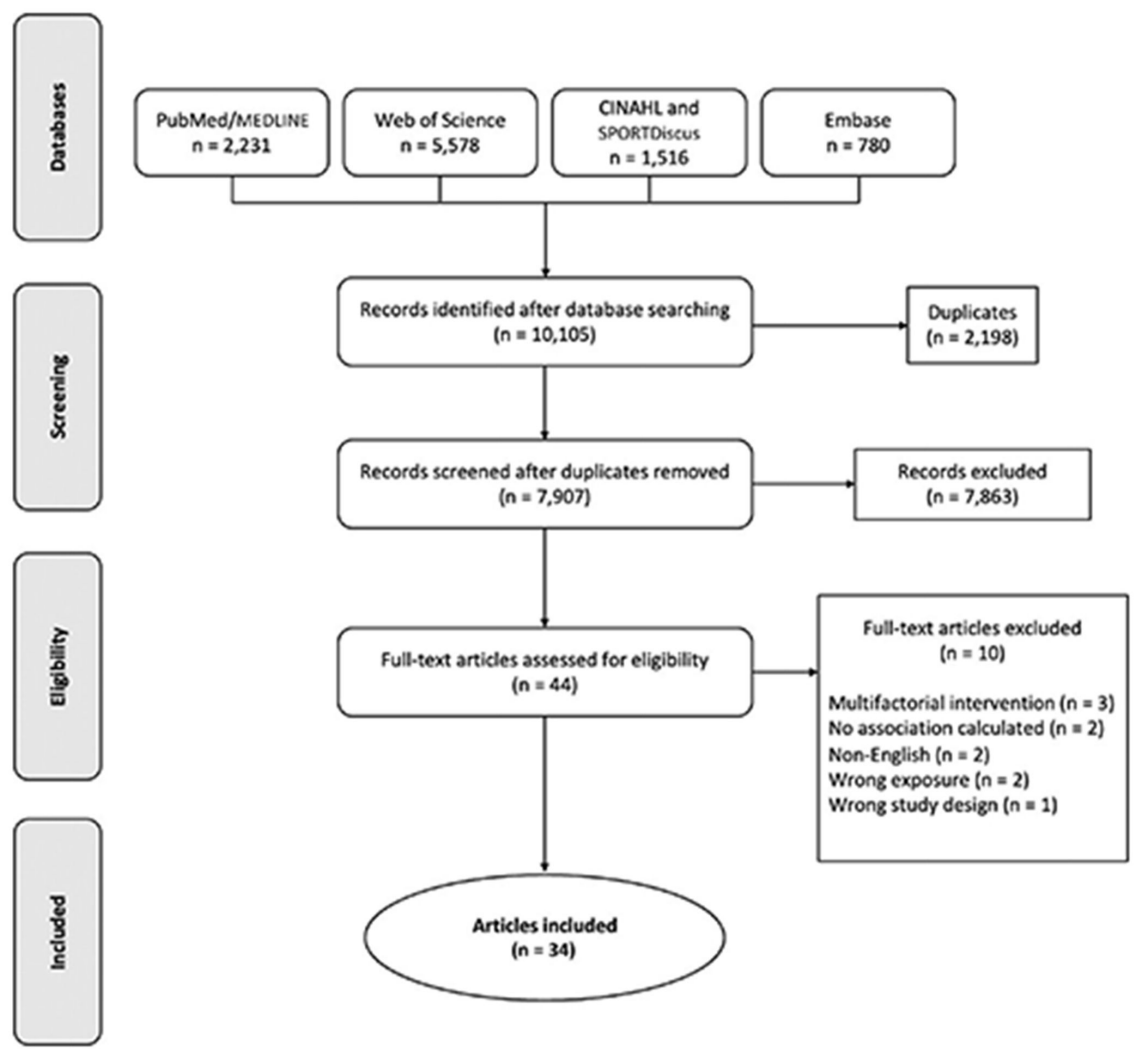


The electronic literature search yielded 10,105 titles, of which 2,198 were removed as duplicates (Figure 1). The remaining 7,907 paper titles and abstracts were examined, 44 were read in full. After applying the inclusion and exclusion criteria, this review included 34 studies from 31 unique populations. Multiple publications came from three study populations including the second^23,24^ and third generation^25^ of the US Nurses’ Health Study and from a cross-sectional Iranian sample.^26,27^ Many of the studies used several different measures of physical activity and will be discussed several times in the results section according to their different physical activity measures. We identified a wide range of potential confounders that were accounted for in multivariable models (Table 2), including sociodemographic characteristics (i.e., race, age, marital status, and education), medical history (i.e., age at menarche, cycle irregularity, and parity), health behaviors (i.e., diet, smoking, and alcohol intake), and other physical behaviors (i.e., sedentary behavior and vigorous physical activity). Descriptive details of each study and their participants included in this review are summarized in Tables 1–3 with additional details described in Supplementary Tables 4 and 5.

### Spontaneous Female Fertility, Physical Activity, and Sedentary Behavior

#### Study characteristics

Twenty-five studies from 22 unique populations reported on the association between physical activity or sedentary behavior and spontaneous fertility among females. Eighteen studies were published in 2011 or later,^16,17,25–40^ 5 between 2000-2010,^23,24,41–43^ and 2 prior to 2000.^44,45^ The study designs were primarily cohort (n=10)^17,24,25,28,30,31,33,35,42,44^ and cross-sectional (n=10),^26,27,32,34,36,37,39–41,43^ along with 4 case control^16,29,38,45^ and 1 case cohort study^23^ (Table 1). Studies were conducted in Asia,^17,26,27,32,34,38,39^ Australia,^28^ Europe,^16,30,35–37,42–44^ North America,^23–25,31,33,40,41,45^ and South America.^29^ Sample size varied by study: 9 studies had a sample of less than 1,000 participants,^16,17,29,30,34,38,41,44,45^ 8 studies had a sample ≥1,000 and less than 3,000 participants,^25–27,31,33,36,39,40^ 5 studies had a sample ≥3,000 and less than 5,000 participants,^32,35,37,42,43^ and 3 studies had a sample ≥5,000 participants.^23,24,28^

#### Exposure: Physical activity and sedentary behavior measurement

All of the studies used subjective measures of physical activity. Nine of 25 studies subjectively assessed physical activity using a self-administered questionnaire,^16,23–25,33,35,36,42,43^ 11 used a self-reported questionnaire,^17,26–32,34,37,40^ and 5 studies used an interviewer-administered questionnaire.^38,39,41,44,45^ Some studies asked participants to recall their current physical activity,^27,37^ or to recall their behaviors within the past 7 days,^16,17,26,29,31,34,38,39^ the past year,^23,24,33,35,41,43,45^ on a typical day,^25,44^ or during a typical week.^28,30,32,36,40,42^ Seventeen of the studies assessed the physical activity exposure based on frequency,^16,17,25,26,28–34,36–39,42,43^ 20 based on duration,^16,17,23–26,28,29,31–36,38–40,42,43,45^ and 19 based on intensity.^16,17,23,24,26,28,29,31–36,38–40,42,44,45^ In addition, 1 study assessed whether the participant was *currently exercising*,^27^ and 1 study assessed physical activity by measuring exercise units^41^ (further detail for these measures of exercise units were not provided).

Four studies created measures of physical activity level (e.g., high, moderate, low) based on reported duration, intensity, and frequency.^32,33,42,45^ The criteria for these physical activity levels were defined by the authors to fit their data and varied by study. Eight studies used their physical activity data to create dichotomous measures assessing adherence to physical activity guidelines (e.g., ≥150 minutes of physical activity per week versus <150 minutes), level (e.g., very active versus inactive), frequency (e.g., everyday versus never), and intensity (e.g., exercise to exhaustion versus easy).^16,27,29,30,37,40,42,43^

Twelve of the 25 studies used Metabolic Equivalent of Task (MET) values assigned to specific activities or large groups of activities.^16,17,23,24,26,28,31,33–35,38,39^ All of the estimated MET values came from the Compendium of Physical Activities.^46^ The MET values assigned to each activity type were then multiplied by the duration/frequency of each activity to provide a total volume indicator of physical activity, accounting for intensity (e.g., MET-min/wk).

Only 7 of 25 studies measured sedentary behavior. Sedentary behavior was measured using either self-administered^16,33^, self-reported^26,28,31,37^, or interviewer administered questionnaires.^38^ Some studies asked participants to recall their typical sedentary behavior,^28,37^ or to recall their behaviors within the past 7 days,^16,26,31,38^ or the past year.^33^ Six of the 7 studies that assessed sedentary behavior used duration of daily sitting as their exposure,^16,26,28,31,33,38^ while 1 study defined sedentary behavior as participant report of *often* or *always* sitting at work.^37^

#### Outcome: Fertility measurement

Most studies (n=22/25) relied on self-reported fertility or infertility status, including: not conceiving after either one-year^16,26,28,32,38,39,41–43^ or six-months,^37^ infertility problems or disorders,^23,24,27^ time to pregnancy,^17,36^ fecundability or fecundability ratio (probability of conception within one menstrual cycle),^31,33,35,44^ current or previous pregnancy or birth,^16,30,34^ and duration of ongoing pregnancy attempt.^25^ The remainder of the studies (n=3/25) relied on clinical measures of fertility including a prospective cohort study that determined fecundability using a urine hCG test,^31^ and 2 case control studies, one that used birth and medical records to identify either primary (not previously conceived) or secondary (had previously conceived) infertility,^45^ and another that used clinical history to identify women who were being treated for anovulatory infertility.^29^

#### Quality assessment

Many of the studies on physical activity, sedentary behavior, and spontaneous female fertility contained an objective that was clearly stated, a defined study population-including pre-specified inclusion and exclusion criteria, well-defined exposure measures and outcome measures, and analyses that adjusted for confounding variables (Supplementary Tables 1 and 2). Few studies provided power calculations, sample size justification, or a description of how participants were selected from eligible recruits. The physical activity exposure was typically only assessed once, and it was generally not assessed prior to assessment of the fertility outcome. Only 2 studies reported if the outcome assessors were blinded to the exposure status of study participants.

#### Findings

##### Female fertility and physical activity level

Upon evaluation of 4 studies that employed measures of physical activity level, 1 study found high levels of physical activity were associated with infertility,^42^ 2 studies found high levels of physical activity were associated with increased fertility^32^ or fecundability,^33^ and 1 study found no association between high levels of vigorous physical activity (e.g., ≥60 minutes/day) and primary or secondary infertility.^45^

Of the 4 studies evaluating physical activity level and female fertility, 3 assessed the associations between moderate levels of physical activity and female fertility. One study found no association,^42^ while 2 studies found that compared to low physical activity levels, moderate levels were associated with a decreased risk of fertility problems.^32,45^

Of the 4 studies evaluating physical activity level and female fertility, none of the studies collected physical activity data at more than one time point. Three of the 4 studies measured the physical activity exposure prior to assessment of the fertility outcome;^33,42,45^ however, only one study was able to ensure temporality.^45^

##### Female fertility and dichotomous measures of physical activity

Among the 8 studies that created dichotomous measures of physical activity, there were no meaningful relationships between measures of physical activity sufficiency (e.g., ≥150 minutes of physical activity per week versus <150 minutes)^16,40,43^ or level (e.g., very active versus inactive).^27,29,37^ The 2 studies that used dichotomous measures of physical activity frequency found conflicting results, one identified a relationship between engaging in ≥3 days per week of recreational physical activity, versus <3 days, and a decreased likelihood of subfertility,^30^ while the other found that exercising almost every day, versus never, was associated with an increased likelihood of infertility.^42^ Lastly, 1 study found that women who reported engaging in high intensity physical activity, versus low intensity, had higher odds of infertility.^42^

None the 8 studies evaluating dichotomous measures of physical activity and female fertility evaluated physical activity at more than one time point. Only one of the studies measured the physical activity exposure prior to assessment of the fertility outcome,^42^ and only one study was able to ensure temporality.^29^

##### Female fertility and physical activity intensity

Among the 12 studies that used MET values, duration, and frequency to account for intensity and volume of physical activity, 7 of the 12 studies used the International Physical Activity Questionnaire (IPAQ) to assign MET values to broad groups of activities (i.e., walking, moderate, or vigorous),^16,17,26,31,34,38,39,47^ 1 of the 12 used a questionnaire similar to the IPAQ that asked participants to report on broad categories of physical activity (i.e., hours per week of vigorous or moderate activity),^35^ while 4 of the 12 studies used questionnaires to assess frequency/duration of specific modes of physical activity (i.e., leisure, occupational, transportation, etc.).^23,24,28,33^ Of those 4 studies that assessed specific modes of physical activity, 1 study asked participants to report on leisure and household physical activity only^23^, 1 study asked participants to report on transportation and leisure physical activity only^28^, 1 study asked participants to report on leisure and occupational physical activity only^24^, and 1 study asked participants to report on leisure, household, occupational, and transportation physical activity.^33^

Among the 12 studies using MET values, 7 reported their associations between the estimated MET values themselves and female fertility. Two of the 7 studies found that high MET-hours/week or MET-minutes/week was associated with decreased fertility^34^ and reduced fecundability^35^, 1 study found higher MET-min/week of vigorous activity was associated with increased fertility^38^, and four studies were unable to identify any meaningful relationships between MET-hours/week or MET-minutes/week and female fertility.^16,17,26,33^

All of the 12 studies that used MET values reported on measures of absolute intensity (e.g., moderate or vigorous) based on estimated MET values and fertility. Ten of the 12 studies evaluated moderate intensity physical activity and female fertility. Seven of the 10 studies did not find any meaningful associations between moderate intensity physical activity and female fertility.^16,23,26,31,33,35,38^ Eleven of the 12 studies evaluated vigorous intensity physical activity and female fertility. Four of the 11 studies did not find any meaningful associations between vigorous intensity or high levels of physical activity and female fertility,^16,17,24,26^ 5 studies found vigorous intensity or higher levels of physical activity were associated with increased fertility^23,28,38^ or fecundability^31,33^ (although McKinnon et al. only observed this relationship among women with a BMI ≥25 kg/m^2^), and 1 study found higher levels of vigorous physical activity were associated with reduced fecundability.^35^ One study found the distribution of high, moderate, and low activity was significantly different between infertile and fertile women, however the study did not report on differences within each level of intensity.^34^

Of the 12 studies evaluating physical activity intensity (based on MET values) and female fertility, 2 studies collected physical activity data at more than one time point.^23,24,28^ Six of the 12 studies measured the physical activity exposure prior to assessment of the fertility outcome.^17,23,24,31,33,35^ Only one study was able to ensure temporality.^23^

##### Female fertility and occupational physical activity

Four out of the 25 studies aimed to assess the association between measures of occupational physical activity and female fertility. One study found increased frequency of handling loads ≥25 kg was associated with a longer duration of pregnancy attempt,^25^ while another study found no association.^37^ Similarly, there was no association between handling loads ≥5 kg and female fertility,^37^ or frequency of walking/standing at work.^25^ Two studies assessed energy expenditure during the workday by measuring fatigue, 1 study found higher levels of fatigue was associated with reduced fecundability,^44^ while the other study found no relationship between frequency of tiredness due to occupational activity and fertility.^42^ In addition to the variation in measures of occupational physical activity, none of the 4 studies measured the occupational physical activity exposure at more than 1 timepoint.

##### Female fertility and walking

Five out of 25 studies evaluated the association between daily walking duration and female fertility. Three studies did not find any meaningful associations,^16,25,26^ one study found increased walking (MET-minutes/week) was associated with increased infertility,^38^ and another study found increased daily walking duration was associated with increased fecundability among women with a body mass index ≥ 25 kg/m^2^.^31^

Among the 5 walking and female fertility studies, only one was able determine if the physical activity exposure occurred prior to the fertility outcome.^31^ Temporality was not addressed, as all 5 studies only measured physical activity at one time point.

###### Female fertility and sedentary behavior

Five of the 7 studies that evaluated the association between sedentary behavior and female fertility did not find any meaningful associations.^26,28,31,33,37^ Two studies found increased sitting time was associated with an increased risk of female infertility.^16,38^ Regarding timing of measurement, only 1 measured sedentary behavior more than once ^28^ and in two studies it was unclear whether the sedentary behavior exposure occurred before or after the female fertility outcome.^16,28^

### Spontaneous Male Fertility, Physical Activity, and Sedentary Behavior

#### Study characteristics

Eleven studies from 11 unique populations reported on the association between physical activity or sedentary behavior and spontaneous fertility among males. Nine studies were published in 2011 or later,^16,17,48–54^ 1 between 2000-2010,^55^ and 1 prior to 2000.^56^ The study designs were primarily randomized controlled trials,^48–52^ along with 3 case control,^16,54,55^ 2 cross-sectional studies^53,56^ and 1 cohort study^17^ (Table 1). Studies were conducted in Asia,^17,48–52,55^ Europe,^16,53,54^ and North America.^56^ Sample size varied by study: 9 studies had a sample of less than 1,000 participants^16,17,48–52,54–56^ and 1 study had a sample ≥5,000 participants.^53^

##### Exposure: Physical activity and sedentary behavior measurement

Six of the studies subjectively assessed physical activity using a self-reported questionnaire,^16,17,53–56^ whereas the physical activity exposure from randomized controlled trials was based on completion of a researcher-administered exercise session.^48–52^ Only 2 studies measured sedentary behavior. To do so, researchers used a self-administered^16^ and self-reported questionnaire.^55^ Some studies asked participants to recall their physical activity and/or sedentary activity behaviors within the past 7 days,^16,17,53,55^ while others ask participants to report their average activity during the past year.^54,56^ Aside from the randomized controlled trials that relied on prescribed exercise sessions as their exposure, 4 of the studies assessed measures of physical activity frequency,^16,54–56^ 4 assessed duration,^16,53,54,56^ and 4 assessed intensity.^16,17,54,55^ Both studies that assessed sedentary behavior used duration of daily sitting as their exposure.^16,55^

#### Outcome: Fertility measurement

Most studies relied on self-reported fertility or infertility based on a one-year definition.^16,17,54^ Or, prior physician diagnosis of infertility,^53^ infertility problems or disorders (male or female partner),^55^ previous or current pregnancy,^16,48–52^ time to pregnancy,^17^ and birth rate.^48–52^

#### Quality assessment

Among the cohort and cross-sectional studies on physical activity, sedentary behavior, and spontaneous male fertility, they all contained an objective that was clearly stated, subjects that were recruited from similar populations, pre-specified inclusion and exclusion criteria, a sufficient time frame to observe an association, and clearly defined physical activity exposures and fertility outcomes measured consistently across participants. None of studies provided the rate of participation among eligible participants and only 1 study included power calculations or sample size justification.^17^ None of the studies measured physical activity more than once and only 1 study assessed physical activity prior to assessment of the fertility measure^17^ (Supplementary Table 1). Among case control and case cohort studies, studies used concurrent controls and the method of processes used to select cases and controls were valid, reliable, and consistent. Additionally, cases were clearly defined and differentiated from controls. None of the case control or case cohort studies included sample size justifications and investigators were unable to confirm that the physical activity exposure occurred prior to the fertility outcome that defined a participant as a case. Studies did not report if the investigators were blinded to the case/control status of participants (Supplementary Table 2). Among the controlled intervention studies, randomization methods were adequate, control and intervention groups were similar at baseline, overall dropout rate was less than 20%, differential dropout rate was less than 15%, and other interventions aside from the study protocol were avoided. Participants and investigators were not blinded to their treatment group in any of the interventions and results were not analyzed using an intention-to-treat analysis (Supplementary Table 3).

#### Findings

##### Male fertility and physical activity level

Three case control, 2 cross-sectional, and 1 cohort study assessed the relationship between physical activity level and male fertility status. Out of the 6 studies, one investigated duration of weekly cycling, finding that among male cyclists, higher duration of weekly cycling was associated with a lower prevalence of infertility.^53^ Two studies found lower levels of past year^54^ and past week^16^ moderate-to-vigorous physical activity were associated with male infertility, while the others found no meaningful relationship between physical activity level and male fertility status.^17,55,56^

Of the 5 studies evaluating physical activity level and male fertility, the largest was from Hollingworth et al. with over 5,000 participants. None of the studies collected physical activity data at more than one time point or were able to ensure temporality.

##### Male fertility and occupational physical activity

Only 1 out of the 10 studies assessed the association between occupational physical activity and male fertility. There was no association between level of physical efforts during work and fertility.^55^ The occupational physical activity exposure was only measured at 1 timepoint.

##### Male fertility and leisure time physical activity interventions

The ability for male physical activity to promote fertility has been investigated through 5 randomized controlled trials of men with idiopathic infertility who did not currently engage in regular physical activity. Compared to men with idiopathic infertility, who maintained a sedentary lifestyle, men, diagnosed with idiopathic infertility, who engaged in aerobic and/or resistance physical activity 3 times per week for 6 months had a higher likelihood of pregnancy up to three months after the intervention, and subsequently a higher live birth rate.^48–52^

Among the 5 randomized controlled trials, neither the participants nor the researchers were blinded in any of the studies. In all 5 studies, the control and the intervention group were similar at baseline. Only 2 of the 5 studies reported high adherence to the treatment protocol.^49,50^

##### Male fertility and sedentary behavior

There was no meaningful association between sedentary behavior and male fertility observed within the 2 included studies.^16,55^ Both studies on sedentary behavior and male fertility were case-control, with less than 200 participants in each, and only one adjusted for potential confounding variables in the analysis.^16^

## Discussion

This systematic review evaluated the associations between physical activity and both female and male spontaneous fertility. There were mixed associations between spontaneous fertility and physical activity, with minimal information regarding spontaneous fertility and sedentary behavior. Based on these results, there is currently insufficient evidence to determine whether physical activity or sedentary behavior is associated with spontaneous female or male fertility.

### Female Fertility and Physical Activity

The evidence for the association between physical activity and female fertility is mixed. For a woman to conceive without artificial methods, her body must be able to regulate the hormones required for ovulation to occur. Physical activity, in combination with other psychosocial and metabolic stressors, can induce a physiological stress response, thereby inhibiting production of estrogen and progesterone, key hormones for ovulation and conception.^57^ It is possible that high levels of physical activity among females could result in an energy deficit, inhibit the processes required for ovulation to occur, and ultimately hinder one’s fertility.^58^ On the other hand, research has also suggested regular, moderate physical activity may aid in achieving optimal hormonal balance and regular ovulation^59^, thus enhancing fertility. However, most of this research has been conducted in overweight and obese women only, citing improved insulin sensitivity and reduction of visceral fat and triglycerides as potential mechanisms between physical activity and improved fertility.^59^ The studies in this review were unable to confirm either of these proposed relationships. Most found mixed results, which is likely for two reasons. First, as discussed earlier, factors that influence fertility can be multi-factorial. Therefore, assessing one aspect of female health (i.e., physical activity), excludes other potentially important lifestyle factors such as diet, alcohol use, or environmental exposures.^60^ Second, ovarian development occurs during gestation when the woman is still a fetus, indicating that a woman’s fertility status is likely due to an accumulation of environmental and physical exposures throughout her lifespan.^61^ The majority of the studies in this review only assessed self-reported female physical activity at one time point.

### Male Fertility and Physical Activity

Results from randomized controlled trials included in this review suggest consistent physical activity among previously sedentary males is associated with increased fertility.^48–52^ Physical activity may increase male fertility by improving sperm parameters necessary for spontaneous conception including sperm concentration and motility.^8^ These results may also signal to the potential for physical activity to serve as a mediator between psychological stress or anxiety and fertility. High levels of stress and anxiety can decrease male testosterone levels leading to a halt in spermatogenesis.^62^ Given that physical activity has been shown to decrease stress and anxiety levels, it is possible that anxious or stressed males may be able to minimize their symptoms through physical activity, which could improve their fertility. However, more research is required and future randomized controlled trials should aim to address this relationship by evaluating methods used to decrease stress and anxiety (e.g., yoga and meditation) to determine if the observed relationship between physical activity and male fertility can be attributed to a reduction in psychological stress.

### Fertility and Sedentary Behavior

Based on limited evidence (n=7), sedentary behavior does not appear to be associated with male or female fertility. However, the sample size of available studies was small and the measurement of sedentary behavior in all of the studies relied on brief self-report measures. Sedentary behavior would be better assessed using accelerometry.^63,64^ It is possible that obesity mediates the relationship between sedentary behavior and fertility. Sedentary behavior is directly associated with BMI which is associated with decreased fertility among both males and females.^4,65^ Future studies should aim to disentangle this relationship in order to determine if sedentary behavior has a direct effect on male and female fertility.

### Strengths and Limitations

To our knowledge, this is the first review to assess the associations between both physical activity and sedentary behavior with spontaneous fertility. Several narrative reviews have been identified which describe the association of physical activity on infertility,^60,66–73^ and 3 meta-analyses have been published - 2 on physical activity and fertility treatment outcomes^74,75^, and one on physical activity and pregnancy rates^76^- yet none of the meta-analyses have discussed sedentary behavior and no systematic reviews have been found on the topic. The findings are the result of a thorough, rigorous review process. Despite its strengths, this review has limitations. First, we only included articles that were published in English. Second, given the variation in how the exposures were measured (5 studies used an interviewer administered questionnaire, 9 used a self-administered questionnaire, 15 used a self-reported questionnaire, and 5 used researcher-administered exercise sessions) and defined, we determined that combining the various measures of physical activity would provide a biased estimate of the relationship between physical activity and fertility, so we were unable to perform meta-analytic analyses to assess for heterogeneity or publication bias.

Aside from the limitations of the review process itself, the included studies also had several limitations. All of the studies used subjective measures of physical activity and sedentary behavior that may be prone to misclassification and response bias. Fertility status was also self-reported in most of the studies included in this analysis, introducing outcome misclassification bias, potentially biasing results away from the null. Finally, most of the studies were cross-sectional, inhibiting researchers from establishing any type of causal relationships between physical activity or sedentary behavior and fertility.

## Conclusions

This systematic review of 34 studies demonstrated there is insufficient evidence to determine the relationship between physical activity and spontaneous female and male fertility. Male participation in physical activity interventions may improve fertility, but future studies should seek to corroborate these relationships. No clear associations were observed between sedentary behavior and spontaneous female or male fertility. In order to yield clinically relevant research, future studies seeking to evaluate the relationships between female or male physical activity, sedentary behavior, and fertility should a) use device-measured methods, in addition to self-report, to measure physical activity and sedentary behavior, such as accelerometry; b) aim to collect measures of physical activity at multiple timepoints during the preconception period, with measures of physical activity and sedentary behavior collected prior to an infertility or subfertility diagnosis; and c) explore potential confounders, effect modifiers, and mediators of physical activity and sedentary behavior on fertility to provide a better understanding of the mechanisms underlying these associations.

## Supplementary Material

Supplementary Material

Supplementary Table S1

Supplementary Table S2

Supplementary Table S3

Supplementary Table S4

Supplementary Table S5

## Figures and Tables

**Table 1:** Descriptive Characteristics of the Studies Included in the Systematic Review on Physical Activity, Sedentary Behavior, and Spontaneous Fertility^a^

Study characteristics	Female fertility studies (n = 25)	Male fertility studies (n = 11)
Study type, n		
Case–cohort	1	--
Case–control	4	3
Cohort	10	1
Cross-sectional	10	2
Randomized controlled trial	--	5
Location, n		
Asia	7	7
Australia	1	--
Europe	8	3
North America	8	1
South America	1	--
Sample size, n		
<1000	9	10
≥1000–<3000	8	--
≥3000–<5000	5	--
≥5000	3	1
Exposure, n		
Physical activity only	18	9
Physical activity and sedentary behavior	7	2
Included multivariable adjustment for potential confounders^b^, n	20	2

a 34 studies were included in this systematic review; however, the studies add to 36 (25 female studies + 11 male studies) because 2 studies evaluated both female and male fertility.

b Key confounders adjusted for vary by study. Some examples include age, marital status, education level, income, diet, and physical activity level.

**Table 2:** Summary of Physical Activity Results From Systematic Review

Author, year of publication Country	Study design	Participant age, y	Participant education level	Participant body mass index, kg/m^2^	Type of physical activity assessed	Physical activity assessment mode^a^	Fertility assessment mode	Confounders included in adjusted models^b^	Physical activity and fertility association^c^
Female fertility and physical activity (n = 25)^d^									
Green et al^26^ United States	Case–control	20–39	Not reported	Not reported	Leisure	Interviewer-administered questionnaire	Birth/medical records	1, 2, 3, 4, 5, 6	Mixed
Florack et al^27^ Netherlands	United States	18–39	<10 y: 35% >14 y: 35%	Not reported	Occupational	Interviewer-administered questionnaire	Interviewer-administered questionnaire	0	Mixed
Rich-Edwards et al^21^ United States	Case–cohort	31.5 (3.6)	Not reported	Not reported	Leisure	Self-administered questionnaire	Self-administered questionnaire	3, 7, 8, 9, 10, 11, 12	Mixed
Chavarro et al^22^ United States	Cohort	25-42	Not reported	23.7 to 24.1	Leisure	Self-administered questionnaire	Self-administered questionnaire	3, 7, 8, 9, 10, 11, 12, 13, 14, 15	No associatio n
Wellons et al^28^ United States	Cross-sectional	Ever infertile: 40.0 (2.9) Never infertile: 40.3 (3.0)	<4 y of college Ever infertile: 61% Never infertile: 44%	≥30: Ever infertile: 39% Never infertile: 31%	Leisure	Interviewer-administered questionnaire	Self-administered questionnaire	1, 3, 9	Mixed
Gudmundsd ottir et al^29^ Norway	United States	20–35	<10 y: 28% 10–12 y:59% >12 y: 13%	Not reported	Leisure and occupational	Self-administered questionnaire	Self-administered questionnaire	3, 8, 11, 16	Mixed
Revonta et al^30^ Finland	Cross-sectional	≥20	Average of 9.1–14.9 y	Average of 24.2 to 28.5	Leisure	Self-administered questionnaire	Interviewer-administered questionnaire	2, 3, 17, 18	No association
Burdorf et al^31^ Netherlands	Cross-sectional	<35: 84% ≥35: 16%	Bachelor’ s degree or higher: 46%	Not reported	Occupatio nal	Selfreported questionnaire	Selfreported questionnaire	3, 18	Mixed
Wise et al^32^ Denmark	United States	27.8–28.9	>4 y of higher education: 24%	Average of 22.9 to 24.9	Leisure	Self-administered questionnaire	Self-administered questionnaire	3, 7, 10, 11, 12 19, 20, 21, 22, 23, 24	Mixed
Esmaeilzad eh et al^25^ Iran	Cross-sectional	≤35: 60%	>12 y: 23%	≥30: 34%	Leisure	Selfreported questionnaire	Selfreported questionnaire	3, 10, 11, 12, 15, 18, 25, 26	No association
Mutsaerts et al^33^ Netherlands	Cross-sectional	30 (4.1)	Vocationa l education or university: 45%	25 (4.7)	Leisure	Self-administered questionnaire	Self-administered questionnaire	0	No association
Esmaeilzad eh et al^24^ Iran	Cross-sectional	33.7 (6.9)	10.2 (4.2) y	27.6 (4.8)	Any	Selfreported questionnaire	Selfreported questionnaire	2, 3, 11, 12, 21, 27, 28, 29	No association
Gaskins et al^23^ United States and Canada	United States	<30: 18% 30-37: 59% >37: 23%	Not reported	≥30: 22%	Occupational	Self-administered questionnaire	Self-administered questionnaire	1, 3, 11, 12, 16, 25, 30	Mixed
Khosrorad et al^34^ Iran	Cross-sectional	Infertile: 30.4 (5.1) Fertile: 28.9 (5.5)	Collegiate Infertile: 31% Fertile: 33%	Not reported	Any	Selfreported questionnaire	Selfreported questionnaire	0	Unfavorable
McKinnon et al^35^ United States and Canada	United States	29.7	Average of 15.9 y	≥30: 23%	Any	Self-administered questionnaire	Self-administered questionnaire	1, 3, 5, 7, 8, 10, 11, 12, 16, 18, 20, 21, 24	Mixed
Cong et al^36^ China	Cross-sectional	Fertile: 37.5 (8.2) Infertile: 38.5 (7.8)	Not reported	Fertile: 23.5 (2.7) Infertile: 23.6 (3.3)	Leisure	Selfreported questionnaire	Selfreported questionnaire	2, 3, 8, 12, 25, 27, 31, 32, 33, 34, 35	Favorable
Russo et al^37^ United States	United States	.-hCG: 29.0 (51) + hCG: 28.7 (4.6)	More than high school, -hCG: 82% +hCG: 89%	-hCG: 27.9 (7.1) +hCG: 25.5 (6.1)	Any	Selfreported questionnaire	hCG^e^ detected pregnancy	8, 12, 16, 36	Mixed
Foucaut et al^38^ France	Case– control	Fertile: 32.2 (3.1) Infertile: 31.1 (4.1)	University or equivalent, Fertile: 87% Infertile: 58%	Fertile: 27.9 (7.1) Infertile: 25.5 (6.1)	Any	Self-administered questionnaire	Self-administered questionnaire	3, 18, 31, 37	No association
Tabernero-Rico et al^39^ Spain	United States	Subfertile: 32.9 (4.1) Nonsubfertile: 30.8 (4.18)	Not reported	≥25: 42%	Leisure	Self-reported questionnaire	Self-reported questionnaire	12	Favorable
Fichman et al^40^ Brazil	Case–control	Infertile: 31 Fertile: 27	Not reported	≥30 Infertile: 50% Fertile: 21%	Any	Selfreported questionnaire	Selfreported questionnaire	0	No association
Mena et al^41^ Australia	United States	22–27	More than high school: 31%	≥30: 5%	Leisure	Self-reported questionnaire	Self-reported questionnaire	2, 3, 16, 18	Favorable
Lam et al^42^ Asia	United States	31.8 (IQR: 29.7–34.1)	Tertiary or above: 88%	≥30: 2%	Any	Self-reported questionnaire	Self-reported telephone interview	0	No association
Dhair et al^43^ Asia	Case–control	30.2 (5.5)	>12 y Infertile: 59% Fertile: 55%	≥30: 21%	Any	Interviewer-administered questionnaire	Interviewer-administered questionnaire	3, 5, 27, 38,39	Favorable
Mirzaei et al^44^ Asia	Cross-sectional	20–29: 27% 30–39: 36% 40–49: 38%	More than high school: 52%	≥30: 27%	Any	Interviewer-administered questionnaire	Interviewer-administered questionnaire	3, 12, 18, 40	Mixed
Shirazi and Rosinger^45^ United States	Cross-sectional	Infertile: 34.4 (0.3) Live birth: 36.0 (0.2)	More than high school Infertile: 79% Live birth: 74%	≥30 Infertile: 39% Live birth: 38%	Aerobic	Self-reported questionnaire	Self-reported questionnaire	0	No association
Male fertility and physical activity (n = 11)^f^									
Baker et al^46^ United States	Cross-sectional	Not reported	More than high school: 100%	Not reported	Leisure	Self-reported questionnaire	Unknown	0	No association
Sheiner et al^47^ Israel	Case-control	Cases: 34.1 (6.2) Controls: 34.4 (7.0)	Cases: 13.2 (2.7) y Controls: 13.1 (3.0) y	Not reported	Occupational	Self-reported questionnaire	Physician reported	0	No association
Ausmees et al^48^ Estonia	Case-control	Cases: 50.0 (IQR: 49.0–54.5) Controls: 53.0 (IQR: 49.0–56.3)	“High” Cases: 49% Controls: 51%	Cases: 27.1 (IQR: 24.9–29.3) Controls: 26.8 (IQR: 24.7–29.5)	Any	Self-reported questionnaire	Self-reported questionnaire	0	Favorable
Hollingworth et al^49^ United Kingdom	Cross-sectional	48.2 (16–88)	Not reported	25.3	Leisure	Self-reported questionnaire	Self-reported questionnaire	3, 10, 11, 12, 41, 42	Mixed
Hajizadeh Maleki et al^50^ Iran	Randomized controlled trial	Asthenozoospermic Ex: 31.7 (8.4) Nonex: 32.6 (7.2) Asthenoteratozoospermic Ex: 33.0 (6.9) Nonex: 32.1 (8.0) Oligospermic Ex: 31.9 (8.2) Nonex: 33.1 (6.4) Oligoasthenozoospermic Ex: 32.6 (8.1) Nonex: 33.8 (6.5) Oligoasthenoteratozoospermic Ex: 34.0 (5.9) Nonex: 32.9 (7.4)	Not reported	Asthenozoospermic Ex: 26.7 (6.2) Nonex: 26.6 (7.4) Asthenoteratozoospermic Ex: 26.3 (5.9) Nonex: 27.1 (6.8) Oligospermic Ex: 27.2 (6.8) Nonex: 27.3 (5.4) Oligoasthenozoospermic Ex: 27.2 (6.8) Nonex: 27.0 (7.1) Oligoasthenoteratozoospermic Ex: 26.7 (6.5) Nonex: 27.0 (8.1)	Leisure	Researcher administered exercise sessions	Birth/medical records	0	Favorable
Hajizadeh Maleki et al^51^ Iran	Randomized controlled trial	Asthenozoospermic Ex: 33.7 (6.3) Nonex: 32.5 (7.4) Asthenoteratozoospermic Ex: 32.1 (7.8) Nonex: 31.5 (8.4) Oligospermic Ex: 34.2 (5.6) Nonex: 33.6 (6.3) Oligoasthenozoospermic Ex: 33.3 (6.6) Nonex: 32.8 (7.0) Oligoasthenoteratozoospermic Ex: 32.8 (7.2) Nonex: 33.2 (6.8)	Not reported	Asthenozoospermic Ex: 27.5 (4.4) Nonex: 27.0 (3.6) Asthenoteratozoospermic Ex: 27.6 (5.0) Nonex: 26.9 (4.7) Oligospermic Ex: 27.1 (5.9) Nonex: 27.5 (6.4) Oligoasthenozoospermic Ex: 27.6 (5.6) Nonex: 27.4 (5.8) Oligoasthenoteratozoospermic Ex: 27.4 (5.6) Nonex: 26.9 (4.5)	Leisure	Researcher administered exercise sessions	Birth/medical records	0	Favorable
Maleki et al^52^ Iran	Randomized controlled trial	Asthenoteratozoospermic Ex: 31.8 (6.7) Nonex: 32.6 (7.1) Oligospermic Ex: 32.1 (7.0) Nonex: 34.0 (5.7) Oligoasthenozoospermic Ex: 31.0 (5.9) Nonex: 32.1 (6.8) Oligoasthenoteratozoospermic Ex: 33.5 (6.3) Nonex: 32.7 (6.4)	Not reported	Asthenoteratozoospermic Ex: 27.6 (5.2) Nonex: 28.1 (6.4) Oligospermic Ex: 28.4 (5.4) Nonex: 27.3 (5.8) Oligoasthenozoospermic Ex: 27.6 (4.0) Nonex: 27.5 (4.4) Oligoasthenoteratozoospermic Ex: 27.0 (3.2) Nonex: 27.7 (4.5)	Leisure	Researcher administered exercise sessions	Birth/medic al records	0	Favorable
Maleki et al^53^ Iran	Randomized controlled trial	Asthenoteratozoospermic Ex: 33.4 (6.1) Nonex: 33.0 (6.8) Oligospermic Ex: 33.5 (6.2) Nonex: 32.8 (6.9) Oligoasthenozoospermic Ex: 34.4 (5.1) Nonex: 32.9 (7.0) Oligoasthenoteratozoospermic Ex: 32.1 (7.9) Nonex: 33.0 (7.2)	Not reported	Asthenoteratozoospermic Ex: 27.8 (7.9) Nonex: 27.3 (6.6) Oligospermic Ex: 27.5 (5.9) Nonex: 27.5 (7.1) Oligoasthenozoospermic Ex: 27.4 (4.2) Nonex: 27.1 (8.0) Oligoasthenoteratozoospermic Ex: 27.8 (4.4) Nonex: 27.4 (6.3)	Leisure	Researcher administered exercise sessions	Birth/medical records	0	Favorable
Foucaut et al^38^ France	Case–control	Fertile: 34.3 (3.9) Infertile: 33.4 (5.3)	University or equivalent Fertile: 81% Infertile: 58%	≥30 Fertile: 33% Infertile: 56%	Any	Self-administered questionnaire	Self-administered questionnaire	3, 18, 31, 36, 37	Mixed
Hajizadeh Maleki et al^54^ Iran	Randomized controlled trial	Asthenozoospermic Ex: 32.4 (7.1) Nonex: 33.6 (6.2) Asthenoteratozoospermic Ex: 31.8 (6.7) Nonex: 32.6 (7.1) Oligospermic Ex: 32.1 (7.0) Nonex: 34.0 (5.7) Oligoasthenozoospermic Ex: 31.0 (5.9) Nonex: 32.1 (6.8) Oligoasthenoteratozoospermic Ex: 33.5 (6.3) Nonex: 32.7 (6.4)	Not reported	Asthenozoospermic Ex: 27.3 (5.7) Nonex: 27.4 (5.5) Asthenoteratozoospermic Ex: 27.6 (5.2) Nonex: 28.1 (6.4) Oligospermic Ex: 28.4 (5.4) Nonex: 27.3 (5.8) Oligoasthenozoospermic Ex: 27.6 (4.0) Nonex: 27.5 (4.4) Oligoasthenoteratozoospermic Ex: 27.0 (3.2) Nonex: 27.7 (4.5)	Leisure	Researcher administered exercise sessions	Birth/medical records	0	Favorable
Lam et al^42^ Asia	United States	33.5 (IQR: 30.3–35.9)	Tertiary or above: 85%	≥30: 7%	Any	Self-reported questionnaire	Self-reported telephone interview	0	No association

Abbreviations: Ex, exercise group; IQR, interquartile range; Nonex, nonexercise group. Note: Age and BMI are mean (SD) or percent.

a Category of physical activity assessment mode was extracted directly from each study.

b Confounders included in adjusted models include (0) final model did not adjust for confounding; (1) race; (2) region; (3) age; (4) date case began trying to conceive or date control successfully conceived; (5) income; (6) number of past sexual partners; (7) moderate physical activity; (8) parity; (9) oral contraceptive use; (10) alcohol intake; (11) smoking; (12) body mass index; (13) calendar time; (14) coffee intake; (15) diet; (16) marital status; (17) university hospital region; (18) education; (19) partner’s age; (20) frequency of intercourse; (21) last method of contraception; (22) cycle length; (23) cycle irregularity; (24) vigorous physical activity; (25) occupation; (26) long-term health problems; (27) age of marriage; (28) history of sexually transmitted diseases; (29) pelvic inflammatory disease; (30) environmental exposures including radiation, antineoplastic drugs, high-level disinfectants, and anesthesia gas; (31) physical activity level; (32) menstruation flow; (33) male staying up late at night; (34) number of abortions; (35) marriage age limit; (36) sedentary behavior; (37) body fat and fat-free mass; (38) refugee status; (39) age of menarche.

c Favorable indicates physical activity exposure was associated with increased fertility outcome; unfavorable indicates physical activity exposure was associated with decreased fertility outcome; mixed indicates the study contained multiple physical activity exposures, and each one had a different association with the fertility outcome; no association indicates no significant relationship between physical activity exposure and fertility outcome.

d See Supplementary Table S1 (available online) for more detailed information.

e Human chorionic gonadotropin.

f See Supplementary Table S2 (available online) for more detailed information.

**Table 3:** Summary of Sedentary Behavior Results From Systematic Review

Author, year of publication, country	Study design	Participant age, y	Participant education level	Participant body mass index, kg/m^2^	Type sedentary behavior assessed	Sedentary behavior assessment mode^a^	Fertility assessment mode	Confounders included in adjusted models^b^	Sedentary behavior and fertility association^c^
Female fertility and sedentary behavior (n = 7)^d^									
Burdorf et al^31^ Netherlands	Cross-sectional	<35: 84% ≥35: 16%	Bachelor’s degree or higher: 46%	Not reported	Sitting	Self-reported questionnaire	Self-reported questionnaire	3, 18	No association
Esmaeilzadeh et al^24^ Iran	Cross-sectional	33.7 (6.9)	10.2 (4.2) y	27.6 (4.8)	Sitting	Self-reported questionnaire	Self-reported questionnaire	2, 3, 11, 12, 21, 27, 28, 29	No association
McKinnon et al^35^ United States and Canada	Cohort	29.7	Average of 15.9 y	≥30: 23%	Sitting	Self-administered questionnaire	Self-administered questionnaire	1, 3, 5, 7, 8, 10, 11, 12, 16, 18, 20, 21, 24	No association
Russo et al^37^ United States	Cohort	-hCG: 29.0 (5.1) +hCG: 28.7 (4.6)	More than high school -hCG: 82% +hCG: 89%	-hCG: 27.9 (7.1) +hCG: 25.5 (6.1)	Sitting	Self-reported questionnaire	hCG^e^ detected pregnancy	8, 12, 16, 36	No association
Foucaut et al^38^ France	Case–control	Fertile: 32.2 (3.1) Infertile: 31.1 (4.1)	University or equivalent Fertile: 87% Infertile: 58%	Fertile: 27.9 (7.1) Infertile: 25.5 (6.1)	Sitting	Self-administered questionnaire	Self-administered questionnaire	3, 18, 31, 37	Unfavorable
Mena et al^41^ Australia	Cohort	22 to 27	More than high school: 31%	≥30: 5%	Sitting	Self-reported questionnaire	Self-reported questionnaire	2, 3, 16, 18	No association
Dhair et al^43^ Asia	Case–control	30.2 (5.5)	>12 y Infertile: 59% Fertile: 55%	≥30: 21%	Sitting	Interviewer-administered questionnaire	Interviewer-administered questionnaire	3, 5, 27, 38, 39	Unfavorable
Male fertility and sedentary behavior (n = 2)^f^									
Sheiner et al^47^ Israel	Case–control	Cases: 34.1 (6.2) Controls: 34.4 (7.0)	Cases: 13.2 (2.7) y Controls: 13.1 (3.0) y	Not reported	Sitting	Self-reported questionnaire	Physician reported	0	No association
Foucaut et al^38^ France	Case–control	Fertile: 34.3 (3.9) Infertile: 33.4 (5.3)	University or equivalent Fertile: 81% Infertile: 58%	≥30 Fertile: 33% Infertile: 56%	Sitting	Self-administered questionnaire	Self-administered questionnaire	3, 18, 31, 36, 37	No association

Note: Age and BMI are mean (SD) or percent.

a Category of sedentary behavior assessment mode was extracted directly from each study.

b Confounders included in adjusted models include (0) final model did not adjust for confounding; (1) race; (2) region; (3) age; (4) date case began trying to conceive or date control successfully conceived; (5) income; (6) number of past sexual partners; (7) moderate physical activity; (8) parity; (9) oral contraceptive use; (10) alcohol intake; (11) smoking; (12) body mass index; (13) calendar time; (14) coffee intake; (15) diet; (16) marital status; (17) university hospital region; (18) education; (19) partner’s age; (20) frequency of intercourse; (21) last method of contraception; (22) cycle length; (23) cycle irregularity; (24) vigorous physical activity; (25) occupation; (26) long-term health problems; (27) age of marriage; (28) history of sexually transmitted diseases; (29) pelvic inflammatory disease; (30) environmental exposures including radiation, antineoplastic drugs, high-level disinfectants, and anesthesia gas; (31) physical activity level; (32) menstruation flow; (33) male staying up late at night; (34) number of abortions; (35) marriage age limit; (36) sedentary behavior; (37) body fat and fat-free mass; (38) refugee status; (39) age of menarche.

c Unfavorable indicates sedentary behavior exposure was associated with decreased fertility outcome; no association indicates no significant relationship between sedentary behavior exposure and fertility outcome.

d See Supplementary Table S1 (available online) for more detailed information.

e Human chorionic gonadotropin.

f See Supplementary Table S2 (available online) for more detailed information.
