# Supplementary material for: Physical activity and fertility
Source: J Phys Act Health. Author manuscript; Available in PMC 2023 Jul 19. (PMC7614776; doi:10.1123/jpah.2022-0487)
Supplement: Supplementary Material [file EMS176148-supplement-Supplementary_Material.pdf]

Supplementary Material. Search Strategy

A. **PubMed Search Strategy:** ((("physical activity" [tiab] OR exercise[tiab] OR sports[tiab] OR "leisure time"[tiab] OR "recreational activity"[tiab] OR "active commuting"[tiab] OR sedentary[tiab] OR sitting[tiab] OR "physical exertion"[tiab] OR "Exercise"[Mesh] OR "Sports"[Mesh] OR "Sedentary Behavior"[Mesh] OR "Sitting Position"[Mesh])) AND ((infertil*[tiab] OR fecund*[tiab] OR ovulation[tiab] OR fertil*[tiab] OR "conception"[tiab] OR "Subfecundity" OR "paternal" OR "Fertility"[Mesh] OR "Infertility"[Mesh])) NOT ("Animals"[Mesh] NOT ("Animals"[Mesh] AND "Humans"[Mesh])))
   Filters: English, Clinical Trial, Editorial, Journal Article, Letter, Meta-Analysis, Observational Study, Randomized Controlled Trial, Review, Systematic review
B. **Web of Science Search Strategy:** (((TI=("physical activity" OR exercise OR sports OR "leisure time" OR "recreational activity" OR "active commuting" OR sedentary OR sitting OR "physical exertion" OR Exercise OR Sports OR "Sedentary Behavior" OR "Sitting Position")) OR (AB=("physical activity" OR exercise OR sports OR "leisure time" OR "recreational activity" OR "active commuting" OR sedentary OR sitting OR "physical exertion" OR Exercise OR Sports OR "Sedentary Behavior" OR "Sitting Position"))) AND ((TI=(infertil* OR fecund* OR ovulation OR fertil* OR conception OR "Subfecundity" OR "paternal" OR Fertility OR Infertility)) OR (AB=(infertil* OR fecund* OR ovulation OR fertil* OR conception OR "Subfecundity" OR "paternal" OR Fertility OR Infertility))))
   Filters: English, Article, Proceedings Paper, Review, Editorial Material, Letter
C. **Embase Search Strategy:** ('physical activity':ti,ab OR exercise:ti,ab OR sports:ti,ab OR 'leisure time':ti,ab OR 'recreational activity':ti,ab OR 'active commuting':ti,ab OR sedentary:ti,ab OR sitting:ti,ab OR 'physical exertion':ti,ab OR 'exercise'/exp OR 'physical activity'/exp OR 'sport'/exp OR 'sedentary lifestyle'/exp OR 'sitting'/exp) AND (infertil*:ti,ab OR fecund*:ti,ab OR ovulation:ti,ab OR fertil*:ti,ab OR conception:ti,ab OR 'subfecundity' OR 'paternal' OR 'fertility'/exp OR 'infertility'/exp) NOT ([animals]/lim NOT [humans]/lim) AND [embase]/lim NOT ([embase]/lim AND [medline]/lim)
   Filters: Article, Review, Editorial, Letter, Article In Press, Short Survey
D. **CINAHL Plus Search Strategy:** ((TI "physical activity" OR AB "physical activity" OR TI exercise OR AB exercise OR TI sports OR AB sports OR TI "leisure time" OR AB "leisure time" OR TI "recreational activity" OR AB "recreational activity" OR TI "active commuting" OR AB "active commuting" OR TI sedentary OR AB sedentary OR TI sitting OR AB sitting OR TI "physical exertion" OR AB "physical exertion" OR (MH "Exercise+") OR (MH "Sports+") OR (MH "Lifestyle, sedentary+") OR (MH "Sitting+"))) AND ((TI infertil* OR AB infertil* OR TI fecund* OR AB fecund* OR TI ovulation OR AB ovulation OR TI fertil* OR AB fertil* OR TI conception OR AB

conception OR TI "paternal" OR AB "paternal" OR TI "Subfecundity" OR AB "Subfecundity" OR (MH "Fertility+") OR (MH "Infertility+")))
Filters: English, Academic Journal

E. **SPORTDiscus Search Strategy:** ((TI "physical activity" OR AB "physical activity" OR TI exercise OR AB exercise OR TI sports OR AB sports OR TI "leisure time" OR AB "leisure time" OR TI "recreational activity" OR AB "recreational activity" OR TI "active commuting" OR AB "active commuting" OR TI sedentary OR AB sedentary OR TI sitting OR AB sitting OR TI "physical exertion" OR AB "physical exertion" OR (MH "Exercise+") OR (MH "Sports+") OR (MH "Lifestyle, sedentary+") OR (MH "Sitting+"))) AND ((TI infertil* OR AB infertil* OR TI fecund* OR AB fecund* OR TI ovulation OR AB ovulation OR TI fertil* OR AB fertil* OR TI conception OR AB conception OR TI "paternal" OR AB "paternal" OR TI "Subfecundity" OR AB "Subfecundity" OR (MH "Fertility+") OR (MH "Infertility+")))
Filters: English, Academic Journal
