# Supplementary material for: Physical activity and fertility
Source: J Phys Act Health. Author manuscript; Available in PMC 2023 Jul 19. (PMC7614776; doi:10.1123/jpah.2022-0487)
Supplement: Supplementary Table S1 [file EMS176148-supplement-Supplementary_Table_S1.pdf]

Supplementary Table S1. Quality assessment ratings for cohort and cross-sectional studies included in the systematic review in publication date order[a]

Year	Author	Q1	Q2	Q3	Q4a	Q4b	Q5	Q6	Q7	Q8	Q9a	Q9b	Q10	Q11a	Q11b	Q12	Q13	Q14
Female fertility outcome																		
1994	Florack	Y	Y	Y	Y	Y	N	Y	Y	Y	Y	Y	N	Y	Y	Y	Y	Y
2007	Chavarro	Y	Y	NR	Y	Y	N	Y	Y	Y	Y	Y	Y	Y	Y	NA	NR	Y
2008	Wellons	Y	Y	Y	Y	Y	N	N	Y	N	N	Y	N	N	Y	NA	NA	Y
2009	Gumundsdottir	Y	Y	Y	Y	Y	N	Y	Y	Y	Y	Y	N	Y	Y	NA	N	Y
2010	Revonta	Y	Y	NR	Y	Y	N	N	NR	N	N	Y	N	N	Y	NA	NA	N
2011	Burdorf	Y	Y	Y	Y	Y	N	N	Y	N	Y	Y	N	Y	Y	NA	NA	Y
2012	Wise	Y	Y	Y	N	Y	N	Y	Y	Y	Y	Y	N	Y	Y	NA	Y	Y
2012	Mutsaerts	Y	Y	Y	Y	Y	N	N	Y	Y	Y	Y	N	Y	Y	NA	NA	Y
2012	Esmaeilzadeh	Y	Y	Y	Y	Y	Y	N	Y	N	Y	Y	N	Y	Y	NA	NA	Y
2013	Esmaeilzadeh	Y	Y	Y	Y	Y	Y	N	Y	Y	Y	Y	N	Y	Y	NA	NA	Y
2015	Gaskins	Y	Y	Y	N	Y	N	Y	Y	Y	Y	Y	N	Y	Y	NA	NR	Y
2015	Khosrorad	Y	Y	NR	Y	Y	Y	N	NR	Y	Y	Y	N	N	Y	NA	NA	N
2016	Cong	Y	Y	NR	Y	Y	NR	N	NA	Y	Y	Y	N	Y	Y	NA	NA	N
2016	McKinnon	Y	Y	Y	N	Y	N	Y	Y	Y	Y	Y	N	Y	Y	NR	N	Y
2018	Russo	Y	Y	Y	Y	Y	N	Y	Y	Y	Y	Y	N	Y	Y	NR	NA	Y
2019	Tabernero-Rico[b]	Y	Y	Y	N	Y	Y	N	N	Y	Y	Y	N	N	Y	NA	NA	Y
2020	Mena	Y	Y	Y	Y	Y	N	N	N	Y	Y	Y	Y	Y	Y	NA	NR	Y
2020	Lam	Y	Y	NR	Y	Y	Y	Y	Y	N	Y	Y	N	Y	Y	NA	Y	N
2020	Mirzaei	Y	Y	NR	Y	Y	N	N	N	Y	Y	Y	N	Y	Y	NA	NA	Y
2021	Shirazi	Y	Y	NR	Y	Y	N	N	N	N	Y	Y	N	Y	Y	NA	NA	Y
**Total "Yes"**		**20**	**20**	**13**	**16**	**20**	**5**	**8**	**13**	**14**	**18**	**20**	**2**	**16**	**20**	**1**	**3**	**16**
Male fertility outcome																		
1988	Baker	Y	Y	NR	Y	Y	N	N	Y	N	Y	Y	N	Y	Y	NA	NA	N
2014	Hollingworth	Y	N	NR	Y	Y	N	N	Y	Y	Y	Y	N	Y	Y	NA	NA	Y
2020	Lam	Y	Y	NR	Y	Y	Y	Y	Y	N	Y	Y	N	Y	Y	NA	Y	N

**Total "Yes"**	**3**	**2**	**0**	**3**	**3**	**1**	**1**	**3**	**1**	**3**	**3**	**0**	**3**	**3**	**0**	**1**	**1**

[a]Quality of included studies was assessed using the National Institutes of Health Study Quality Assessment Tool for Observational Cohort and Cross-Sectional Studies (http://www.nhlbi.nih.gov/health-pro/guidelines/in-develop/cardiovascular-risk-reduction/tools/cohort). **Q1**: Was the research question or objective in this paper clearly stated?; **Q2**: Was the study population clearly specified and defined?; **Q3**: Was the participation rate of eligible persons at least 50%?; **Q4a**: Were all subjects selected or recruited from the same or similar populations (including the same time period)?; **Q4b**: Were inclusion and exclusion criteria for being in the study prespecified and applied uniformly to all participants?; **Q5**: Was a sample size justification, power description, or variance and effect estimates provided?; **Q6**: Were the exposures of interest measured prior to the outcomes being measured?; **Q7**: Was the timeframe sufficient so that one could reasonably expect to see an association between the exposure and outcome if it existed?; **Q8**: For exposures that can vary in amount or level, did the study examine different levels of the exposure as it related to the outcome?; **Q9a**: Were the exposure measures clearly defined?; **Q9b**: Were the exposure measures implemented consistently across all study participants?; **Q10**: Was the exposure assessed more than once over time?; **Q11a**: Were the outcome measures clearly defined?; **Q11b**: Were the outcome measures implemented consistently across all study participants?; **Q12**: Were the outcome assessors blinded to the exposure status of participants?; **Q13**: Was loss to follow-up after baseline 20% or less?; **Q14**: Were potential confounding variables measured and adjusted statistically for their impact on the relationship between the exposure and outcome? Y, yes; N, No; NR, not reported; NA, not applicable.

[b]Study enrolled two groups of women into an observational study: a subfertile group referred after an infertility consultation and a pregnant group attending their first prenatal appointment. The authors described the study as a "prospective comparative study with two groups". We evaluated the quality based on the cohort study assessment.
