# Supplementary material for: Physical activity and fertility
Source: J Phys Act Health. Author manuscript; Available in PMC 2023 Jul 19. (PMC7614776; doi:10.1123/jpah.2022-0487)
Supplement: Supplementary Table S2 [file EMS176148-supplement-Supplementary_Table_S2.pdf]

Supplementary Table S2. Quality assessment ratings for case control and case cohort studies included in the systematic review by female and male fertility outcome in publication date order[a]

Year	Author	Q1	Q2	Q3	Q4	Q5	Q6	Q7	Q8	Q9	Q10	Q11
Female fertility outcome												
1986	Green	Y	Y	N	Y	N	Y	N	Y	Y	NR	Y
2002	Rich-Edwards	Y	Y	N	Y	Y	Y	Y	Y	Y	NA	Y
2019	Foucaut	Y	Y	N	Y	Y	Y	NA	Y	N	NA	Y
2020	Dhair	Y	Y	Y	Y	Y	Y	N	Y	N	NR	Y
2020	Fichman	Y	Y	Y	Y	Y	Y	NA	Y	Y	N	N
**Total "Yes"**		**5**	**5**	**2**	**5**	**4**	**5**	**1**	**4**	**3**	**0**	**3**
Male fertility outcome												
2002	Sheiner	Y	Y	N	Y	Y	Y	NA	Y	N	N	N
2014	Ausmees	Y	Y	N	N	Y	Y	NA	Y	NR	NA	N
2019	Foucaut	Y	Y	N	Y	Y	Y	NA	Y	N	NA	Y
**Total "Yes"**		**3**	**3**	**0**	**2**	**3**	**3**	**0**	**3**	**0**	**0**	**1**

[a]Quality of included studies was assessed using the National Institutes of Health Study Quality Assessment Tool for Observational Cohort and Cross-Sectional Studies (https://www.nhlbi.nih.gov/health-topics/study-quality-assessment-tools) **Q1**: Was the research question or objective in this paper clearly stated and appropriate?; **Q2**: Was the study population clearly specified and defined?; **Q3**: Did the authors include a sample size justification?; **Q4**: Were controls selected or recruited from the same or similar population that gave rise to the cases (including the same timeframe)?; **Q5**: Were the definitions, inclusion and exclusion criteria, algorithms or processes used to identify or select cases and controls valid, reliable, and implemented consistently across all study participants?; **Q6**: Were the cases clearly defined and differentiated from controls?; **Q7**: If less than 100 percent of eligible cases and/or controls were selected for the study, were the cases and/or controls randomly selected from those eligible?; **Q8**: Was there use of concurrent controls?; **Q9**: Were the investigators able to confirm that the exposure/risk occurred prior to the development of the condition or event that defined a participant as a case?; **Q10**: Were the assessors of exposure/risk blinded to the case or control status of participants?; **Q11**: Were potential confounding variables measured and adjusted statistically in the analyses? If matching was used, did the investigators account for matching during study analysis?; Y, yes; N, No; NR, not reported; NA, not applicable.
