# Supplementary material for: Physical activity and fertility
Source: J Phys Act Health. Author manuscript; Available in PMC 2023 Jul 19. (PMC7614776; doi:10.1123/jpah.2022-0487)
Supplement: Supplementary Table S3 [file EMS176148-supplement-Supplementary_Table_S3.pdf]

Supplementary Table S3. Quality assessment ratings for controlled intervention studies included in the systematic review in publication date order[a]

Year	Author	Q1	Q2	Q3	Q4	Q5	Q6	Q7	Q8	Q9	Q10	Q11	Q12	Q13	Q14
2017	Maleki-*J Obstet Gynaecol Can*	Y	Y	N	N	NR	Y	Y	Y	NR	Y	Y	Y	Y	N
2017	Maleki-*Appl Physiol Nutr Metab*	Y	Y	N	N	NR	Y	Y	Y	Y	Y	Y	Y	Y	N
2017	Maleki-*Cytokine*	Y	Y	N	N	NR	Y	Y	Y	NR	Y	Y	Y	Y	N
2018	Maleki	Y	Y	N	N	NR	Y	Y	Y	Y	Y	Y	Y	Y	N
2020	Maleki	Y	Y	N	N	NR	Y	Y	Y	NR	Y	Y	N	Y	N
**Total "Yes"**		**5**	**5**	**0**	**0**	**0**	**5**	**5**	**5**	**2**	**5**	**5**	**4**	**5**	**0**

[a]Quality of included studies was assessed using the National Institutes of Health Study Quality Assessment Tool for Controlled Intervention Studies (https://www.nhlbi.nih.gov/health-topics/study-quality-assessment-tools) **Q1**: Was the study described as randomized, a randomized trial, a randomized clinical trial, or an RCT?; **Q2**: Was the method of randomization adequate (i.e., use of randomly generated assignment)?; **Q3**: Was the treatment allocation concealed (so that assignments could not be predicted)?; **Q4**: Were study participants and providers blinded to treatment group assignment?; **Q5**: Were the people assessing the outcomes blinded to the participants' group assignments?; **Q6**: Were the groups similar at baseline on important characteristics that could affect outcomes (e.g., demographics, risk factors, co-morbid conditions)?; **Q7**: Was the overall drop-out rate from the study at endpoint 20% or lower of the number allocated to treatment?; **Q8**: Was the differential drop-out rate (between treatment groups) at endpoint 15 percentage points or lower?; **Q9**: Was there high adherence to the intervention protocols for each treatment group?; **Q10**:Were other interventions avoided or similar in the groups (e.g., similar background treatments)?; **Q11**: Were outcomes assessed using valid and reliable measures, implemented consistently across all study participants?; **Q12**: Did the authors report that the sample size was sufficiently large to be able to detect a difference in the main outcome between groups with at least 80% power?; **Q13**: Were outcomes reported or

subgroups analyzed prespecified (i.e., identified before analyses were conducted)?; **Q14**: Were all randomized participants analyzed in the group to which they were originally assigned, i.e., did they use an intention-to-treat analysis?; Y, yes; N, No; NR, not reported;

NA, not applicable.
