# Supplementary material for: Physical activity and fertility
Source: J Phys Act Health. Author manuscript; Available in PMC 2023 Jul 19. (PMC7614776; doi:10.1123/jpah.2022-0487)
Supplement: Supplementary Table S4 [file EMS176148-supplement-Supplementary_Table_S4.pdf]

Supplementary Table S4. Characteristics of the studies included in the systematic review on physical activity, sedentary behavior, and female fertility in date order by year (n = 25)[a,b]

Author, Year; Study Design	Sample Size for Analysis and Country	Years of Data Collection	Description of Sample	PA and Sedentary Exposure	Assessment Mode for PA/ Sedentary Behavior and Outcomes	Outcomes	Potential Confounders	Main Results
Green et al.,1986<br><br>case control	Primary infertility:<br>Cases: 199<br>Controls:199<br><br>Secondary infertility:<br>Cases: 162<br>Controls: 428<br><br>United States	1979-1981	Women, 20-39 years and residents of King County, Washington whose first medical evaluation for infertility occurred during the years 1979-1981	Categories of past year average minutes/day in vigorous exercise: (1) non-exercise; (2) <60 minutes/day (3) ≥60 minutes/day	PA: interviewer administered questionnaire<br><br>Outcomes: birth record and medical record	Women defined as being infertile (those who did not conceive despite trying for at least one year) in whom an ovulatory abnormality was present. Primary infertility (not previously conceived) and secondary infertility (had previously conceived) also explored.	Confounders considered: Race, census tract of residence, age, reference date (date case began trying to conceive and date control successfully conceived), socioeconomic factors (income, education, social class of partner/participant occupation), past birth control use, number of past sexual partners, drug and smoking habits, history of sexually transmitted diseases, body weight at the time of successful or failed conception, weight relative to height, percent of weight under or over ideal body weight, and, occupational exertion<br><br>Confounders included in adjusted models:<br>Race, census tract of residence, age, reference date (date case began trying to conceive and date control successfully conceived), income, and number of past sexual partners	Among nulligravid women, past year vigorous exercise for ≥60 minutes/day was not associated with infertility (ARR 1.9, 90% CI 0.6, 5.1), while exercising <60 minutes/day was associated with a lower risk (ARR 0.6, 90% CI 0.4, 0.9), both compared to not exercising. There was no association found among women who were previously pregnant.

Florack et al., 1994<br><br>cohort	260<br><br>Netherlands	1987-1989	Female workers, 18-39 years working in nonmedical functions at 39 Dutch hospitals	Total energy expenditure during a usual working day referred to a fatigue score	PA: interviewer administered questionnaire<br><br>Outcomes: interviewer administered questionnaire	Fecundability was defined as the probability of becoming pregnant per month (time to pregnancy estimated as the number of months between the date of starting unprotected intercourse and a positive pregnancy test).	Confounders considered: Age, previous pregnancies, chronic disease, regular drug use, occupational exposures (detergents, vibration, video display terminals), and characteristics of the partner (smoking, alcohol, caffeine intake)<br><br>Final models did not adjust for confounders	A high fatigue score was associated with reduced fecundability compared to women with a low fatigue score among all women (UOR 0.6, 95% CI 0.4, 1.0) and among women with unfavorable working hours (UOR 0.4, 95% CI 0.2, 0.8). Moderate fatigue scores compared to lower fatigue scores were not associated with fecundability overall or among women with unfavorable working hours.
Rich-Edwards et al., 2002<br><br>case cohort	Cases: 830<br><br>Controls: 26,125<br><br>United States	1989-1995	Nurses were enrolled in the Nurses' Health Study II who were 25-42 years at cohort inception in 1989	Past year vigorous LTPA (hours/wk) in seven categories; moderate LTPA (hours/wk) in four categories	PA: self-administered questionnaire<br><br>Outcomes: self-administered questionnaire	Cases were defined as self-reported ovulatory disorder Infertility.<br>Controls were pregnancies reported in 1991-1995.	Confounders considered: Age, time spent in moderate PA, parity, recency of oral contraceptive use, alcohol intake, smoking, and BMI<br><br>Confounders included in adjusted models:<br>Age, time spent in moderate PA, parity, recency of oral contraceptive use, alcohol intake, smoking, and BMI	Past year vigorous LTPA was associated with a reduced odds of ovulatory infertility (5-<7 hours/wk AOR 0.66, 95% CI 0.48, 0.92 and ≥7 hours/wk AOR 0.63, 95% CI 0.44, 0.91, both compared to <0.5 hours/wk). Each hour/wk of vigorous LTPA was associated with a lower odds of ovulatory infertility (AOR 0.95, 95% CI 0.92, 0.98). Each hour/wk of moderate LTPA was not associated with ovulatory infertility (AOR 1.01, 95% CI 0.99, 1.03).
Chavarro et al, 2007<br><br>cohort	17,544<br><br>United States	1991-1999 (PA data in 1991 related to 1991-1997 period and PA in 1997	Nurses' Health Study II, started in 1989. Included nurses 25–42 years, married, without a history of infertility, and with available	Past year LTPA (five categories of vigorous PA in minutes/day)	PA: self-administered questionnaire<br><br>Outcomes: self-administered	Self-reported ovulatory infertility and all other causes of infertility (endometriosis, tubal (blockage) factor, male factor, or	Confounders considered: Age, calendar time, parity, smoking, OCP use, alcohol intake, coffee intake<br><br>Confounders included in adjusted models: Age,	Time spent in past year vigorous LTPA was not associated with either ovulatory infertility and all other causes of infertility in adjusted models. The associations were modified by parity (p for interaction 0.03) with an indication

		related to remainder of follow-up)	information on diet, PA, height, and weight		questionnaire	cervical (mucous) factor)	calendar time, alcohol intake, coffee intake, moderate PA, parity, smoking, OCP use, fertility diet score, and BMI	of an inverse association among nulliparous women but not parous women. Vigorous LTPA was not modified by age or BMI.
Wellons et al., 2008<br><br>cross-sectional	489<br><br>United States	1985-2000	Women aged 33–44 years from four communities in the Coronary Artery Risk Development In young Adults (CARDIA) Study (Birmingham, AL; Chicago, IL; Minneapolis, MN; Oakland, CA)	Continuous scores and low LTPA in the past year	PA: interviewer administered questionnaire<br><br>Outcomes: self-administered questionnaire	Self-report of having unprotected intercourse for at least 12 months without becoming pregnant.	Confounders considered: Age, race, and hormonal contraception use<br><br>Confounders included in adjusted models: Age, race, and hormonal contraception use	Ever infertile women had lower past year LTPA scores compared to never infertile women (p=0.02) in unadjusted analyses. No association between LTPA and fertility in adjusted analyses (AOR 1.16, 95% CI 0.95, 1.42).
Gudmundsdottir et al., 2009<br><br>cohort	3,887<br><br>Norway	1984–1986 (HUNT 1) and in the follow-up study in 1995–1997 (HUNT 2)	All women residents ≥ 20 years in the county of Nord-Trøndelag, Norway were invited to participate in the study	Frequency, intensity, and duration of usual week LTPA. Based on frequency, intensity, and duration of usual week LTPA, a PA index was calculated and categorized into high, moderate, and low PA levels.<br><br>Occupational PA was assessed through frequency of feeling tired from occupational work categorized	PA: self-administered questionnaire<br><br>Outcomes: self-administered questionnaire	Women were classified as fertile if they conceived within 1 year of attempting to become pregnant and gave birth, and infertile if they did not conceive within 1 year, regardless of any subsequent pregnancies.	Confounders considered: Age, parity, smoking, frequency of alcohol consumption during the 14 days prior to study participation, marital status, BMI, and education<br><br>Confounders included in adjusted models:<br>Age, parity, smoking, and marital status	Exercising almost every day (e.g., higher frequency) was associated with a higher odds of infertility compared to never exercising (AOR 3.2, 95% CI 1.3, 7.6). Exercising to exhaustion (e.g., intensity) was associated with higher odds of infertility compared to exercising easy (AOR 2.3, 95% CI 1.2, 4.5). Exercising for >60, 30-60, and 16-30 minutes/session (e.g., duration) was associated with a reduced odds of infertility compared to a duration of 0-15 minutes/session (AOR 0.6, 95% CI 0.3, 1.2; AOR 0.5, 95% CI 0.3, 0.9; AOR 0.3, 95% CI 0.2, 0.5, respectively). Based on the PA index, those with high PA levels had higher odds of infertility compared to those with low PA levels (AOR 1.5, 95% CI 1.0, 2.3). There was no association between

				into almost never, seldom, often, and almost always				moderate PA levels and infertility, compared to those with low PA levels (AOR 0.9, 95% CI 0.6, 1.5). Infertile women did not differ from fertile women in regard to frequency of feeling tired from occupational work (chi-square test p=0.097).
Revonta et al., 2010<br><br>cross-sectional	3,049<br><br>Finland	2000-2001	Women aged 20 and over of which the information concerning infertility and other related factors	Sufficient exercise was defined as leisure time activity or commuting to work with bicycle or by walking ≥0.5 hour/day ≥2 times/wk.	PA: self-administered questionnaire<br><br>Outcomes: interviewer-administered questionnaire	Women were defined as infertile if they had reported unsuccessful conceiving ≥1 year and never given birth nor ever had an induced abortion	Confounders considered: Age, place of residence, university hospital region, and education<br><br>Confounders included in adjusted models: Age, place of residence, university hospital region, and education	Infertile women ages 20-34 (66%), 35-49 (72%), and ≥50 years (2%) reported a similar prevalence of sufficient exercise compared to fertile women ages 20-34 (55%), 35-49 (67%), and ≥50 years (13%) (chi-squared test p=0.17, 0.47, 0.58 for ages 20-34, 35-49, and ≥50 years, respectively).
Burdorf et al., 2011<br><br>cross-sectional	3,719<br><br>Netherlands	2002-2006	Pregnant women with a delivery date between April 2002 and January 2006	Physical workload defined as prolonged (often/always) standing at work or manually handling loads ≥5 kg or manually handling loads ≥ 25 kg. Sedentary behavior defined as prolonged (often/always) sitting work.	PA: self-reported questionnaire<br><br>SB: self-reported questionnaire<br><br>Outcome: self-reported questionnaire	Prolonged time to pregnancy (>6 months unprotected intercourse before becoming pregnant) reported when pregnant	Confounders considered: Age, height, weight, education, country of origin, parity, smoking habits, and alcohol use<br><br>Confounders included in adjusted models: Age and education	In univariate analyses, handling loads ≥25 kg (OR 1.17, 95% CI 0.56, 2.44), prolonged sitting (OR 0.90, 95% CI 0.77, 1.04), and prolonged standing (OR 1.01, 95% CI 0.81, 1.25) were not associated with a prolonged time to pregnancy. Handling loads ≥ 5 kg was associated with a decreased likelihood of a prolonged time to pregnancy in univariate analyses (OR 0.74, 95% CI 0.57, 0.97), this association was no longer significant after adjustment (AOR 0.82, 95% CI 0.61, 1.09)
Wise et al., 2012<br><br>cohort	3,628<br><br>Denmark	2007–2009	Women 18–40 years, residents of Denmark, in a stable relationship with a	Total past year LTPA MET hours/wk calculated by	PA: self-administered questionnaire	Assessed fecundability. Women censored if they did not conceive	Confounders considered: Age, partner's age, BMI, alcohol consumption, pack-years of smoking, frequency	Vigorous LTPA was associated with reduced fecundability (≥5 hours/wk vs. none: AFR 0.68, 95% CI 0.54, 0.85), while moderate

			male partner, and not receiving any type of fertility treatment	summing the METS from vigorous (hours/wk multiplied by 7.0) and moderate (hours/wk multiplied by 3.5)	Outcomes: self-administered questionnaire	after 12 cycles. Total cycles at risk calculated as: (days of attempt time at study entry/usual cycle length) + [(last menstrual period date from most recent follow-up questionnaire - date of baseline questionnaire completion)/usual cycle length] +1.	of intercourse, last method of contraception, cycle length, and cycle irregularity, vigorous and moderate LTPA<br><br>Confounders included in adjusted models:<br>Age, partner's age, BMI, alcohol consumption, pack-years of smoking, frequency of intercourse, last method of contraception, cycle length, and cycle irregularity; vigorous LTPA is adjusted for moderate LTPA and vice versa	LTPA was not (≥5 hours/wk vs. <1: AFR 1.18, 95% CI 0.98, 1.43). Higher total MET-hours/wk was associated with reduced fecundability (≥60 AFR 0.74, 95% 0.56, 0.97 vs. 20-29 MET-hours/wk). Inverse associations between high vigorous LTPA and fecundability were observed within subgroups of age, parity, cycle regularity, and <25 kg/m^2 BMI, but no associations were observed among women ≥25 kg/m^2.
Esmaeilzadeh et al., 2012<br><br>cross-sectional	1,081<br><br>Iran	Not mentioned	Women married for at least one year, 20-45 years, mentally sound	Current exercise (yes versus no)	PA: self-reported questionnaire<br><br>Outcomes: self-reported questionnaire	Lifetime infertility problems coded as primary (before birth of a child) or secondary (after birth of 1 or more children)	Confounders considered:<br>Age, education, occupation, partner occupation, long-term health problems, history of tubal surgery, ectopic pregnancy, or chlamydia, IUD use, OCP use, tubal sterilization, BMI, smoking, partner smoking, alcohol, and diet<br><br>Confounders included in adjusted models: Age, education, occupation, partner occupation, long-term health problems, BMI, smoking, partner smoking, alcohol, and diet	Current exercise was not associated with lifetime infertility problems compared to those that did not report exercise (OR 1.20; 95% CI 0.84, 1.70).
Mutsaerts et al., 2012<br><br>cross-sectional	1,924<br><br>Netherlands	2006-2007	All pregnant women with expected date of delivery between April 2006-April	PA level (defined as at least moderate intensity for ≥30	PA: self-administered questionnaire	Time to pregnancy, defined as interval between when the couple set out to	Adjusted analyses were not performed	In bivariate analysis, weekly PA was not associated with time to pregnancy (p=0.52).

			2007 in Drenthe, Netherlands	minutes/day) was subdivided into five weekly categories (no activity, sometimes, 1 time/wk, 2-3 times/wk, and ≥4 times/wk)	Outcomes: self-administered questionnaire	conceive to date of conception measured in months.		
Esmaeilzadeh et al., 2013<br><br>cross-sectional	1,081<br><br>Iran	Not mentioned	Women married for at least one year, 20-45 years, mentally sound	IPAQ-short provided MET-minutes/wk, of vigorous PA, moderate PA, walking, and total PA; PA level (high, moderate, and low); and duration of daily walking. SB was defined as MET-minutes/wk and daily duration of sitting.	PA: self-reported questionnaire<br><br>SB: self-reported questionnaire<br><br>Outcomes: self-reported questionnaire	Infertility (delay in conception for at least 12 months) and lifetime infertility (any past report of infertility)	Confounders considered: Age, smoking, age of marriage, area, past history of sexually transmitted disease, pelvic inflammatory disease, contraception, occupation, and BMI<br><br>Confounders included in adjusted models: age, smoking, age of marriage, area, history of sexually transmitted disease, pelvic inflammatory disease, contraception, occupation, BMI	Infertile women did not differ from fertile women by PA level or MET-minutes/wk of vigorous PA, moderate PA, walking, total PA, or sitting. PA level, daily walking duration, and daily sitting duration were not associated with lifetime infertility.
Gaskins et al., 2015<br><br>cohort	1,739<br><br>United States and Canada	2010-2014	Nurse or nursing student, born on or after January 1965, and trying to get pregnant	Duration (hours/day) of standing/ walking or frequency (times per day) of moving a heavy load (≥25 pounds) while at work.	PA: self-administered questionnaire<br><br>Outcomes: self-administered questionnaire	Women who report that they were actively trying to get pregnant were asked to report the current duration of their ongoing pregnancy attempt.	Confounders considered: Age, race/ethnicity, BMI, lifetime pregnancy history, smoking history, and marital status<br><br>Confounders included in adjusted models: Age, BMI, smoking status, marital status, race, work-related factors, and current exposure to radiation, antineoplastic drugs, high-level disinfectants, and	Duration of standing/walking at work in hours/day was not associated with duration of pregnancy attempt. In fully adjusted models, the frequency of moving or lifting heavy loads (≥25 pounds) at work was associated with a longer duration of pregnancy attempt compared to not lifting at work (>15 times/day: time ratio 1.49, 95% CI 1.20, 1.85; 6-15 times/day: time ratio 1.15, 95% CI 0.99, 1.33; 1-5 times/day:

							anesthesia gas	time ratio 1.13, 95% CI 1.00, 1.28); test for trend=0.002.
Khosrorad et al., 2015<br><br>cross-sectional	216<br><br>Iran	2013-2014	Couples were recruited if they were Iranian, 18-45 years, and literate from an IVF clinic	IPAQ-long provided MET minutes/wk categorized into high, moderate, and low levels of PA.	PA: self-reported questionnaire<br><br>Outcomes: self-reported questionnaire	Couples were considered fertile if the woman had given birth to at least one child (over six months before the study) and had gotten pregnant without ART. Couples who had an infertility diagnosis for more than one year were regarded as infertile.	Adjusted analyses were not performed	Infertile women had higher levels of PA than fertile women (p=0.03): high (>3000 MET-minutes/wk), moderate (600-3000 MET-minutes/wk), and low (<600 MET-minutes/wk) categories for infertile women (20%, 73%, 7%) compared to fertile women (9%, 86%, 5%).
McKinnon et al., 2016<br><br>cohort	2,062<br><br>United States and Canada	2013-2016	21 to 45 years, not using contraception or fertility treatments, in a stable relationship with a male partner, planning a pregnancy, and not currently pregnant could enroll in PRESTO (Boston University Pregnancy Study Online)	Past year moderate PA and vigorous PA (hours/wk) and past year total MET-hours/wk. SB was defined as sitting time (hours/day).	PA: self-administered questionnaire<br><br>SB: self-administered questionnaire<br><br>Outcomes: self-administered questionnaire	Fecundability, measured as time to pregnancy; calculated total cycles at risk from the reported number of cycles trying to conceive at study entry, date of LMP before enrolment, usual cycle length, and LMP date on each follow-up questionnaire. Participants contributed cycles to the analysis from the time of enrolment until reported pregnancy, initiation of fertility treatment, loss to follow-up, or 12 cycles, whichever	Confounders considered: Age, race/ethnicity, education, smoking history, intercourse frequency, last method of contraception, marital status, income, parity, alcohol consumption, and male partner BMI. Vigorous PA and moderate PA were mutually adjusted; analyses of MET were not further adjusted for either type of PA.<br><br>Confounders included in adjusted models: Age, parity, intercourse frequency, education, race/ethnicity, household income, marital status, BMI, last method of contraception, alcohol consumption, smoking, partner BMI, and PA.	Past year moderate PA was associated with higher fecundability in unadjusted models only. After adjustment, past year vigorous PA 3-4 hours/wk was associated with a higher FR compared to <1 hour/wk (AFR 1.16, 95% CI 1.00, 1.35). Past year total MET-hours/wk was not associated with fecundability in adjusted models. Among overweight and obese women, fecundability was higher for vigorous PA ≥5 versus <1 hours/wk (AFR 1.27, 95% CI 1.02, 1.57), but not among normal weight women. Sitting was not associated with fecundability in adjusted models.

						came first.		
Cong et al., 2016<br><br>cross-sectional	4,232<br><br>China	2014	Local residents, aged 20 to 49 years and married	Light exercise (<3 times/wk), regular exercise (>1 hour and at least 3 times/wk), or heavy exercise (intense athletic competition or engaged in athletic related occupations)	PA: self-reported questionnaire<br><br>Outcomes: self-reported questionnaire	Infertility defined as a failure to achieve a clinical pregnancy after ≥12 months of regular unprotected sex.	Confounders considered: BMI, PA level, menstruation flow, male staying up late at night, male engaged in high-temperature occupations, number of pregnancies, number of abortions, age, terrain, age at marriage, and marriage age limit<br><br>Confounders included in adjusted models: BMI, PA level, menstruation flow, male staying up late at night, male engaged in high-temperature occupations, number of pregnancies, number of abortions, age, terrain, age at marriage, and marriage age limit	Infertility incidence was lower among regularly (OR 0.25; 95% CI 0.10, 0.65) and heavy (OR 0.58; 95% CI 0.42, 0.81) exercising women compared to light exercising women.
Russo et al., 2018<br><br>cohort	1,214<br><br>United States	2007-2011	Women, aged 18 40 years, with a history of 1 or 2 miscarriages. All women were participants in the Effects of Aspirin in Gestation and Reproduction (EAGeR) trial.	IPAQ-short provided frequency (days/wk) and duration (minutes/day) of walking, vigorous PA, moderate PA, and sitting. IPAQ-short also provided MET-min/wk categorized into high, medium, or low levels of PA.	PA: self-reported questionnaire<br><br>SB: self-reported questionnaire<br><br>Outcome: hCG detected pregnancy	Fecundability, measured as the number of menstrual cycles to pregnancy	Confounders considered: Marital status, parity, BMI, and sitting<br><br>Confounders included in all adjusted models: Marital status and parity<br><br>Confounders included in models stratified by BMI: Marital status, parity, and BMI<br><br>Confounders included in model stratified by BMI with vigorous PA as primary exposure: Marital status,	Vigorous PA >4 hrs/wk was associated with increased fecundability (AOR 1.69, 95% CI 1.24, 2.31). The association of walking and fecundability differed by BMI categories. Among overweight/obese women (BMI≥30 kg/m^2) walking was associated with increased fecundability (AOR 1.82, 95% CI 1.19, 2.77) compared to overweight/obese women who reported no walking. Walking was not associated with fecundability among normal and underweight women. Fecundability was not associated with moderate PA, total baseline exercise, or sitting.

							parity, BMI, and sitting	
Foucaut et al., 2019<br><br>case control	Cases: 80<br>Controls: 71<br><br>France	2009-2013	Cases: Women, younger than 38 years, had a history of at least 12 months of unprotected sexual intercourse with no diagnosed etiology for infertility. They had no previous history of miscarriages and had not received infertility treatment<br><br>Controls: Women, younger than 38 years, had a recent natural and spontaneous pregnancy and delivery within the last 2 years with a time to conceive that was less than 12 months	IPAQ-short provided 7-day history of frequency (days/wk) and duration (minutes/day) of vigorous PA, moderate PA, and sitting. PA was categorized by adherence to PA guidelines (≥ 150 minutes of moderate to vigorous PA per week), duration of moderate PA (min/wk), duration of vigorous PA (min/wk) total MET-min/wk, and duration of walking (min/wk)	PA: self-administered questionnaire<br><br>SB: self-administered questionnaire<br><br>Outcomes: self-administered questionnaire	Women were considered fertile if they reported a natural and spontaneous pregnancy and delivery within the last 2 years with a time to conception that was less than 12 months	Confounders considered: Age, education level, PA level, sedentary behavior, body fat, fat-free mass, BMI, and waist circumference<br><br>Confounders included in adjusted models: Age, education level, PA level, sedentary behavior, body fat, and fat-free mass	Infertile women did not differ from fertile women by mean physical activity level (MET-min/wk), mean walking time (min/wk), mean vigorous PA (min/wk), mean moderate PA (min/wk) or adherence to physical activity guidelines (≥150 minutes moderate to vigorous PA/ week) (Wilcoxon-Mann-Whitney test p=0.8,0.08, 0.07, 0.5, 0.3). Non-adherence to PA guidelines (<150 min/wk of moderate to vigorous PA) was not associated with infertility (AOR 1.58, 95% CI 0.73, 3.42) compared to women who reported ≥150 minutes of moderate to vigorous PA per week.<br><br>Infertile women did not differ from fertile women by mean sitting time (hours/day) (t-test p=0.9). Typical weekday sitting time ≥5 hours/day was associated with infertility (AOR 3.61, 95% CI 1.58, 8.24) compared to women who reported less than 5 hours of sitting time on a typical weekday.
Tabernero-Rico et al., 2019<br><br>cohort	Cases: 200<br>Controls: 197<br><br>Spain	2009-2013	Subfertile: Nulliparous women, 18-40 years, with a male partner not	Frequency of regular recreational PA with five possible	PA: self-reported questionnaire	Women were considered fertile if they were pregnant with their first child	Confounders considered: Age, ethnicity, presence of associated morbidity, BMI, diet, PA level, education	Women who were subfertile reported recreational PA 3-4 days/wk less often than comparison women (30.6% vs 45.2%, p=0.01).

			receiving assisted reproductive therapy who were referred for an infertility consultation.<br><br>Non-subfertile: Primigravidae women, 18-40 years, at their first gestation consultation.	answers categorized into two levels: 3-4 days recreational PA /wk (yes versus no)	Outcomes: self-reported questionnaire	and attending a gestation consultation	level, income<br><br>Confounders included in adjusted models: BMI	Women who reported PA on 3-4 days/wk had a decreased likelihood of subfertility (AOR 0.33, 95% CI 0.15, 0.71) compared to women who reported less than 3 days/wk.
Fichman et al., 2020<br><br>case control	Cases: 24<br>Controls: 28<br><br>Brazil	2017	Cases:<br>Women, 20-37 years, being treated at the outpatient infertility clinic with known anovulatory problems<br><br>Controls:<br>Pregnant women, 20-37 years, being treated at the prenatal outpatient clinic	IPAQ-short categorized into very active/active and irregularly active/sedentary. Recall period was current period for cases and pregestational period for controls.	PA: self-reported questionnaire<br><br>Outcomes: Clinical history of infertility	Women were considered infertile if they were being treated at the infertility outpatient clinic, with anovulatory problems defined by the physician.	Adjusted analyses were not performed	Infertile women (58%) reported a similar prevalence of very active/somewhat active PA and irregularly active/SB compared to fertile women (61%) (Fisher's exact test p=1.000).
Mena et al., 2020<br><br>cohort	6,130<br><br>Australia	2000-2015	Women born between 1973-1978 and included in the Australian Medicare database. To be included women had to complete the 2000 survey (baseline) and respond to at least 2 out of the 5	Weekly duration of walking, and moderate to vigorous leisure or transportation PA. MET-min/wk were calculated and categorized as high, moderate, low or none total	PA: Self-reported questionnaire<br><br>SB: Self-reported questionnaire<br><br>Outcome: Self-reported questionnaire	Fertility problems, measured as having tried unsuccessfully to get pregnant for ≥12 months	Confounders considered: Age, marital status, country of birth, and highest qualification<br><br>Confounders included in adjusted models: Age, marital status, country of birth, and highest qualification	Compared with women who reported low levels of PA, women with high levels of PA had a decreased risk of fertility problems (AOR 0.82, 95% CI 0.69, 0.98). In women with normal BMI, moderate (AOR 0.74, 95% CI 0.57-0.96), and high levels (AOR 0.64, 95% CI 0.49, 0.82), of PA were associated with a decreased risk of fertility problems compared

			additional surveys between 2000-2015. Additionally, women had to indicate they had tried to conceive or become pregnant on at least 1 of the surveys.	volume of PA. SB was defined as sitting time (Low: <4.5 hours/day, Moderate: ≥4.5- <8 hours/day, or High: ≥ 8 hours/day)				to women with normal BMI who reported low levels of PA. Compared to women with low sitting time, moderate (AOR 0.93, 95% CI 0.79, 1.09) and high sitting time (AOR 1.04, 95% CI 0.86, 1.26) were not associated with risk of fertility problems.
Lam et al., 2020<br><br>cohort	100<br><br>Hong Kong	2015-2019	Nulliparous women aged 20-44 years. Women had to be part of a couple who was planning to conceive (i.e., had stopped contraception for ≤ 6 months or were about to stop contraception). Participants had no prior history of infertility, coital dysfunction, tuboperitoneal disease, pelvic inflammatory disease, ectopic pregnancy, endometriosis, anovulation, irregular menstrual cycles (cycle length <21 or >35 days, endocrine disease, use of hormonal treatment that may affect ovarian function within the	English and Chinese IPAQ-short provided total MET-minutes/wk of PA.	PA: self-reported questionnaire<br><br>Outcomes: telephone interview	Fecundability was defined as time to pregnancy, which was the period from when the couple started to have regular unprotected intercourse without contraception to conception.	Adjusted analyses were not performed	Compared to women with low levels of PA, higher levels of PA (MET-min/wk) were not associated with fecundability (OR 1.00, 95% CI 1.00, 1.00). Female physical activity level was not significantly correlated with the time to pregnancy within one year (Spearman correlation coefficient: 0.24, p=0.091).

			past 3 months, or use of hormonal contraception within the past 6 months.					
Dhair et al., 2020<br><br>Case-control	320<br><br>Palestine	2016-2018	Cases: Married, sexually active women of reproductive age (18-49 years) with a history of at least 12 months of unprotected sexual intercourse with no diagnosed etiology for infertility and had not received infertility treatment<br><br>Controls: Women of reproductive age (18-49 years) who had at least 2 successful pregnancies during their lifetime, with no history of infertility or infertility treatment	IPAQ-short provided 7-day history of frequency (days/wk) and duration (minutes/day) of vigorous PA, moderate PA, and sitting, which were used to calculate MET-minutes/wk, of vigorous PA, moderate PA, walking, and total PA and PA level (high, moderate, and low)<br><br>SB was defined as daily duration of sitting	PA: interviewer-administered questionnaire<br><br>SB: interviewer-administered questionnaire<br><br>Outcomes: interviewer-administered questionnaire	Infertility was defined as having tried unsuccessfully to get pregnant for ≥12 months	Confounders considered: Age, refugee status, residency status, area, location, years of education, employment status, employment status of husband, average monthly income, marital age, age of menarche<br><br>Confounders included in adjusted model: Age, refugee status, marital age, age of menarche, and average monthly income	Compared with women who reported high levels of PA, women with low levels of PA (AOR 3.20, 95% CI 1.55-6.60) or moderate levels of PA (AOR 1.41, 95% CI 0.74-2.70) had an higher odds of infertility. Infertile women (cases) did not differ from fertile women (controls) in regards to moderate PA intensity (MET-min/wk Mann Whitney U test p=0.73) or total PA (MET-min/wk Mann Whitney U test p=0.54). Infertile women (16%) were less likely than fertile women (26%) to practice high intensity PA. Infertile women had lower vigorous PA (MET-min/wk Mann Whitney U test p=0.01) and higher walking PA (MET-min/wk Mann Whitney U test p=0.004).<br><br>Infertile women spent more time sitting (mean 274 minutes/day) compared to fertile women (225 minutes/day). Compared to women who had less than 300 minutes/day of sedentary time, women with ≥300 minutes/day of sedentary time had higher odds of infertility (OR 2.27, 95% CI 1.36-3.79). Longer durations of daily sedentary time were associated with higher odds of infertility (OR 28.2, 95% CI 2.1-54.2).
Mirzaei et al.,	2,611	2014-2015	Local, aged 20 to 49	Persian IPAQ-	PA:	Infertility defined as	Confounders considered:	Infertile women reported less PA

2020<br><br>Cross-sectional	Iran		years, who were participating in the Yazd Health Study	short provided MET minutes/wk categorized into high, moderate, and low levels of PA.	Interviewer administered questionnaire<br><br>Outcomes: Interviewer administered questionnaire	a failure to achieve a clinical pregnancy after ≥12 months of unprotected sex.	Education level, age, BMI, smoking, and waist circumference<br><br>Confounders included in adjusted model: Age, education, BMI, and waist circumference	PA compared to fertile women (Mann-Whitney U test p=0.02).<br><br>The odds of infertility were higher among women with low PA (AOR: 3.51; 95% CI: 3.00, 4.02) and high PA (AOR: 2.01; 95% CI: 1.44, 2.57) compared to women with moderate PA
Shirazi et al. 2020 Cross-sectional	974 (infertility) and 1714 (live birth ratio) United States	2013-2016 for infertility and 2007-2016 for live birth ratio	Women aged 23 to 45 years old who participated in NHANES between 2013 and 2016 were asked a question on infertility and number of live births. For the infertility analysis, women were not pregnant, breastfeeding, or using hormonal contraceptives.	Weekly duration of moderate aerobic PA. PA was categorized by adherence to PA guidelines (≥ 150 minutes/week of moderate aerobic PA	PA: self-reported questionnaire<br><br>Outcomes: self-reported questionnaire	Infertility defined as a failure to achieve a clinical pregnancy after ≥12 months of unprotected sex. Pregnancy rate defined as the number of times a woman reported she had been pregnant. Live birth rate defined as the number of pregnancies that ended in a live birth.	Confounders considered: Age, highest level of education achieved, food security category, health-insurance security, time in the USA, BMI category, smoking, alcohol consumption, diabetes<br><br>Confounders included in adjusted model: Not reported but likely these same variables	Adherence to PA guidelines (≥150 minutes/week of moderate aerobic PA) was not associated with infertility (AOR 1.46, 95% CI: 0.87, 2.47) or live birth ratios (β: 0.02± 0.02) compared to women who reported <150 minutes/week of moderate aerobic PA.

[a]Abbreviations: **A**: adjusted; **ART**: assisted reproductive technology; **BMI**: body index mass; **CI:** confidence interval; **FR**: fecundability ratio; **IPAQ:** International Physical Activity Questionnaire; **IUD**: intrauterine device; **IVF:** in vitro fertilization; **LMP**: last menstrual period; **LTPA**: leisure-time physical activity; **MET**: metabolic equivalent of task; **OCP**: oral contraceptive pill; **OR:** odds ratio; **PA**: physical activity; **RR:** relative risk; **SB:** sedentary behavior; **U**: unadjusted

[b]Physical activity, sedentary behavior, and outcomes were classified as "self-reported" when it was not clear whether the questionnaire was interviewer- or self-administered
