# Supplementary material for: Physical activity and fertility
Source: J Phys Act Health. Author manuscript; Available in PMC 2023 Jul 19. (PMC7614776; doi:10.1123/jpah.2022-0487)
Supplement: Supplementary Table S5 [file EMS176148-supplement-Supplementary_Table_S5.pdf]

Supplementary Table S5. Characteristics of the studies included in the systematic review on physical activity, sedentary behavior, and male fertility in date order by year (n = 11)[a,b]

Author, Year; Study Design	Sample Size for Analysis and Country	Years of Data Collection	Description of Sample	PA and Sedentary Exposure	Assessment Mode for PA/ Sedentary Behavior and Outcomes	Outcomes	Potential Confounders	Main Results
Baker et al., 1988<br><br>cross-sectional	69<br><br>United States	1979-1983	Men, who were not taking any medication, with no history of chronic disease, genitourinary infection, or infertility that were participating in a donor insemination program. Participants were mostly medical students (n=55) or residents, graduate students, or physicians (n=14).	Frequency and duration of past year PA categorized into high (3-4 days per week of 1-2 hours PA sessions and 30 min/day of individual conditioning in either running racquetball, tennis, biking, swimming, soccer, or skiing) and low PA (rarely participated in sports)	PA: Self-reported questionnaire<br><br>Outcome: not known	Pregnancy achieved with their donated sperm	Adjusted analyses were not performed	Compared to participation in low activity, high activity was not associated with decreased pregnancy rates (Fisher's exact test p=0.28). Pregnancy was achieved using sperm from 3% (2/21) high active and 70% (9/48) low active men.
Sheiner et al., 2002<br><br>case control	Cases: 106<br>Controls: 66<br><br>Israel	1999-2000	Cases: Men, who spoke Hebrew and were attending the Soroka University Medical Center because of a male infertility problem.<br><br>Controls: Men, who spoke Hebrew and were attending the Soroka University Medical Center because of a	Weekly frequency of PA (No; once/wk; twice/wk; $\geq$3/wk) and physical efforts during work (difficult, moderate, or mild). SB was defined as duration of sitting time at work (hours/day)	PA: self-reported questionnaire<br><br>SB: self-reported questionnaire<br><br>Outcomes: physician-reported	Men were considered fertile if their physician did not diagnose them with male fertility problems and diagnosed their female partner with fertility problems.	Adjusted analyses were not performed.	Infertile men (cases) did not differ from fertile men (controls) in regards to weekly PA frequency (Fisher's exact test p=0.12), physical efforts during work (chi-square test p=0.99) or mean sitting time at work (t-test p=0.46).

			female infertility problem.					
Ausmees et al., 2014<br><br>case control	Cases: 164<br><br>Controls: 61<br><br>Estonia	Cases: 2000-2010<br><br>Controls: 2007-2010	Cases:<br>Men, greater than 45 years, with known couple infertility who visited the Andrology Center of Tartu University Hospital<br><br>Control: Men who self-reported fatherhood, greater than 45 years, with no history of prior chronic illness or infertility who attended a prostate health screening at the Andrology Center of Tartu University Hospital	Past year moderate-intensity PA ≥30 minutes/day on 5 days of the week (yes, continuously; yes, non-continuously; or no)	PA: Self-reported questionnaire<br><br>Outcomes: Self-reported questionnaire	Men were considered healthy if they had no prior history of chronic illness, continual use of medicine, and history of infertility. Men who had a preceding period of couple infertility (sexually active, noncontraceptive time not resulting in pregnancy) for more than one year were regarded as infertile.	Adjusted analyses were not performed	Infertile men had lower levels of PA than fertile men ($p<0.001$ from chi-square test): Continuously (at least 30 minutes per day for 5 days per week during the past year), non-continuously (at least 30 minutes per day for 5 days per week during the past year) and no (<30 minutes per day for 5 days per week during the past year) categories for infertile men (7%, 12%, 81%) compared to fertile men (20%, 48%, 33%).
Hollingworth et al. 2014<br><br>cross-sectional	5,282<br><br>United Kingdom	2012-2013	All men who saw the online survey advertised in cycling magazines or within UK cycling organizations were invited to participate	Weekly cycling time (hours/wk)	PA: self-reported questionnaire<br><br>Outcomes: self-reported questionnaire	Men were considered infertile if they reported a physician had diagnosed them with infertility	Confounders considered:<br>Age, smoking, weekly alcohol intake, BMI, physician-diagnosed hypertension, and weekly duration of other (non-cycling) physical activities using the IPAQ<br><br>Confounders included in adjusted models:	Cycling 3.75-5.75 hours/wk was associated with decreased odds of infertility (OR 0.44; 95% CI 0.21, 0.94) compared to men who cycled less than 3.75 hours/wk. The other categories were not associated: cycling 5.76-8.5 hours/wk (OR 0.64; 95% CI 0.34, 1.22) and >8.5 hours/wk (OR 0.56; 95% CI 0.28, 1.11). There was no linear association between cycling and infertility (p-trend=0.14).

							Age, smoking, weekly alcohol intake, BMI, physician-diagnosed hypertension, and weekly duration of other (non-cycling) physical activities using the IPAQ	
Maleki et al., 2017<br><br>randomized controlled trial<br><br>*Appl Physiol Nutr Metab*	Intervention: 278<br>Control: 278<br><br>Iran	2012-2013	Married men, aged 25 to 40, attending the infertility clinic where recruitment was conducted. All men had a history of idiopathic infertility (>1 year), had a sedentary lifestyle without a regular exercise program, were non-smokers, and did not have alcohol dependence. All participants' female partners received normal results on a fertility evaluation. Participants were required to cease all medical therapies ≥ 12 weeks before the intervention began. Participants had no prior history of chronic illness, use of antioxidant supplements, irregular eating, mental illness,	Intervention group participated in 24 weeks (3 sessions/wk) of combined aerobic and resistance training (Aerobic: 30-35 min of walking/running on the treadmill; resistance: 30-35 minutes of strength exercises for all major muscle groups)	PA: researcher-administered exercise sessions<br><br>Outcomes: telephone interview and medical record	Pregnancy rate (number of successful pregnancies during and 3 months after the intervention period) and live birth rate (number of pregnancies that ended in a live birth)	Adjusted analyses were not performed	Participating in combined aerobic and resistance training 3 times/wk for 24 weeks was associated with increased odds of pregnancy (OR 97.0, 95% CI 44.6, 211.1) and an increased likelihood of a live birth (OR 20.2, 95% CI 4.4, 92.2) compared to a sedentary control group.

			abnormal development, or occupational exposure that could have reduced reproductive capacity.					
Maleki et al., 2017<br><br>randomized controlled trial<br><br>*Cytokine*	Intervention: 210<br>Control: 209<br><br>Iran	2014	Married men, aged 25 to 40, attending the infertility clinic where recruitment was conducted. All men had a history of idiopathic infertility (>1 year), had a sedentary lifestyle without a regular exercise program (<25 min moderate PA on 3 or more days/wk), were non-smokers, and did not use alcohol 6 months prior to the study. Participants were required to cease all medical therapies ≥ 12 weeks before the intervention began. Participants had no prior history of chronic illness, use of antioxidant supplements, irregular eating, mental illness, abnormal development, or occupational exposure that could have reduced reproductive capacity.	Intervention group participated in 24 weeks of moderate aerobic PA performed on a treadmill (Week 1-12: 3-4 sessions/wk, 35-30 minutes of treadmill running; Week 13-24: 4-6 sessions/wk, 40-45 minutes of treadmill running)	PA: researcher-administered exercise sessions<br><br>Outcomes: telephone interview and medical record	Pregnancy rate (number of successful pregnancies during and 3 months after the intervention period) and live birth rate (number of pregnancies that ended in a live birth)	Adjusted analyses were not performed	Participating in resistance training 3 times/wk for 24 weeks was associated with increased odds of pregnancy (OR 80.0, 95% CI 32.5, 646.2) and an increased likelihood of a live birth (OR 197.0, 95% CI 5.9, 2149.6) compared to a sedentary control group.
Maleki et al., 2017	Intervention: 218	Not stated	Men, aged 25 to 40, attending the infertility	Intervention group participated in 24	PA: researcher-administered	Pregnancy rate (number of	Adjusted analyses were not performed	Participating in high-intensity exercise 3 times/wk for 24 weeks was associated with

randomized controlled trial<br><br>*J Obstet Gynaecol Can*	Control: 215<br><br>Iran		clinic where recruitment was conducted. All men had a history of idiopathic infertility (>1 year), were sedentary (<25 minutes of moderate PA on most days) and had female partners who received normal results on a fertility evaluation. Participants had no prior history of chronic illness, use of antioxidant supplements, use of alcohol or cigarettes in the last 6 months, irregular eating, mental illness, abnormal development, or occupational exposure that could have reduced reproductive capacity	weeks (3 sessions/wk) of high-intensity PA performed on a treadmill (Week 1-12: 40-50 minutes of treadmill running; Week 13-24: 50-60 minutes of treadmill running)	exercise sessions<br><br>Outcomes: telephone interview and medical record	successful pregnancies during and 3 months after the intervention period) and live birth rate (number of pregnancies that ended in a live birth)		increased odds of pregnancy (OR 6.0, 95% CI 2.5, 14.4) and was not associated with an increased likelihood of a live birth (OR 3.3, 95% CI 0.6-17.7) compared to a sedentary control group.
Maleki et al., 2018<br><br>randomized controlled trial	Intervention: 199<br>Control: 208<br><br>Iran	Not reported	Married men, aged 25 to 40, attending the infertility clinic where recruitment was conducted. All men had a history of idiopathic infertility (>1 year), had a sedentary lifestyle without a regular exercise program (<25 min moderate PA/wk), were non-smokers, and did not use alcohol 6 months prior to the	Intervention group participated in 24 weeks (3 sessions/wk) of resistance training (strength exercises for all major muscle groups)	PA: researcher-administered exercise sessions<br><br>Outcomes: telephone interview and medical record	Pregnancy rate (number of successful pregnancies during and 3 months after the intervention period) and live birth rate (number of pregnancies that ended in a live birth)	Adjusted analyses were not performed	Participating in resistance training 3 times/wk for 24 weeks was associated with increased odds of pregnancy (OR 17.7, 95% CI 7.7, 40.7) and an increased likelihood of a live birth (OR 16.2, 95% CI 2.4, 108) compared to a sedentary control group.

			study. All participants' female partners received normal results on a fertility evaluation. Participants were required to cease all medical therapies ≥ 12 weeks before the intervention began. Participants had no prior history of chronic illness, use of antioxidant supplements, irregular eating, mental illness, abnormal development, or occupational exposure that could have reduced reproductive capacity.					
Foucaut et al., 2019<br><br>case control	Cases: 79<br>Controls: 72<br><br>France	2009-2013	Cases: Men, younger than 45 years, had a history of at least 12 months of unprotected sexual intercourse with no diagnosed etiology for infertility. They had not received infertility treatment.<br><br>Controls: Men, younger than 45 years, had a recent natural and spontaneous pregnancy and delivery within the last 2 years with a time to conceive that was less than 12 months.	IPAQ-short provided 7-day history of frequency (days/wk) and duration (minutes/day) of vigorous PA, moderate PA, and sitting. PA was categorized by adherence to PA guidelines (≥ 150 minutes of moderate to vigorous PA per week), duration of moderate PA (min/wk),	PA: self-administered questionnaire<br><br>SB: self-administered questionnaire<br><br>Outcomes: Self-administered questionnaire	Men were considered fertile if they reported a natural and spontaneous pregnancy and delivery within the last 2 years with a time to conception that was less than 12 months	Confounders considered: Age, education level, PA level, sedentary behavior, body fat, fat-free mass, BMI, and waist circumference<br><br>Confounders included in adjusted models: Age, education level, PA level, sedentary behavior, body fat, and fat-free mass	Infertile men did not differ from fertile men by mean physical activity level (MET-min/wk), mean walking time (min/wk), mean moderate PA (min/wk), or adherence to physical activity guidelines (≥150 minutes moderate to vigorous PA/wk) (Wilcoxon-Mann-Whitney test p=0.7, 0.2, 0.7, 0.05). Infertile men reported less vigorous PA compared to fertile men (Wilcoxon-Mann-Whitney test p=0.006). Non-adherence to PA guidelines (<150 min/wk of moderate to vigorous PA) was associated with infertility (AOR 2.20, 95% CI 1.06, 4.58) compared to men who reported ≥150 minutes of moderate to vigorous PA per week.<br><br>Infertile men did not differ from fertile men by mean sitting time (hours/day) (t-

				duration of vigorous PA (min/wk), total MET-min/wk, and duration of walking (min/wk)				test p=0.7). Typical weekday sitting time ≥5 hours/day was not associated with infertility (AOR 1.20, 95% CI 0.55, 2.61) compared to men who reported less than 5 hours of sitting time on a typical weekday.
Maleki et al., 2020<br><br>randomized controlled trial	Intervention: 221<br>Control: 220<br><br>Iran	Not stated	Married men, aged 25 to 40, attending the infertility clinic where recruitment was conducted. All men had a history of idiopathic infertility (>1 year), had a sedentary lifestyle without an exercise program. All participants' female partners received normal results on a fertility evaluation. Participants were required to cease all medical therapies ≥ 12 weeks before the intervention began. Participants had no prior history of chronic illness, use of antioxidant supplements, use of alcohol or cigarettes in the last 3 months, irregular eating, mental illness, abnormal development, or	Intervention group participated in 24 weeks (3 sessions/wk) of high-intensity PA on a treadmill	PA: researcher-administered exercise sessions<br><br>Outcomes: telephone interview and medical record	Pregnancy rate (number of successful pregnancies during and 3 months after the intervention period) and live birth rate (number of pregnancies that ended in a live birth)	Adjusted analyses were not performed	Participating in high-intensity exercise 3 times/wk for 24 weeks was associated with increased odds of pregnancy (OR 4.9, 95% CI 2.0, 11.7) and an increased likelihood of a live birth (OR 9.5, 95% CI 1.3-69.9) compared to a sedentary control group.

			occupational exposure that could have reduced reproductive capacity.					
Lam et al., 2020<br><br>cohort	100<br><br>Hong Kong	2015-2019	Men had to be part of a couple who was planning to conceive (i.e., had stopped contraception for ≤ 6 months or were about to stop contraception). Their female partner had to be nulliparous, aged 20-44 years old. Participants had no prior history of infertility, coital dysfunction, use of hormonal treatment that may affect testicular function within the past 3 months, azoospermia, and medical or genetic diseases that may affect fertility	English and Chinese IPAQ-short provided total MET-minutes/wk of PA.	PA: self-reported questionnaire<br><br>Outcomes: telephone interview	Fecundability was defined as time to pregnancy, which was the period from when the couple started to have regular unprotected intercourse without contraception to conception.	Adjusted analyses were not performed	Compared to men with lower levels of PA, higher levels of PA (MET-min/wk) were not associated with fecundability (OR 1.00, 95% CI 1.00, 1.00). Male physical activity level was not significantly correlated with the time to pregnancy within one year (Spearman correlation coefficient: -0.08, p=0.574).

[a]Abbreviations: **A**: adjusted; **BMI**: body index mass; **CI**: confidence interval; **IPAQ**: International Physical Activity Questionnaire; **MET**: metabolic equivalent of task; **OR**: odds ratio; **PA**: physical activity; **SB**: sedentary behavior

[b]Physical activity, sedentary behavior, and outcomes were classified as “self-reported” when it was not clear whether the questionnaire was interviewer- or self-administered.
